# Innovative sustainable concrete with waste glass materials: an explainable machine learning for compressive strength prediction

**DOI:** 10.1038/s41598-026-64655-w

**Published:** 2026-08-07

**Authors:** Abdelrahman Shams, Suhaib Rasool Wani, Eman Mousa, Abdelrahman Kamal Hamed

**Affiliations:** 1 Civil Engineering Department, Faculty of Engineering, Horus University-Egypt, New Damietta, 34517 Egypt; 2 Advanced Centre of Research and Innovation, CGC University, Mohali, 140307 India; 3 Irrigation and Hydraulics Engineering Department, Faculty of Engineering, Tanta University, Tanta, 31733 Egypt

**Keywords:** Compressive strength, Machine learning, Sustainable concrete, Shapley analysis, LightGBM, Engineering, Materials science

## Abstract

Sustainable concrete incorporating waste glass materials has emerged as a promising solution to reduce environmental impacts associated with cement production and natural aggregate depletion. Accurate prediction of compressive strength (CS) is essential for optimizing such mixtures and ensuring structural reliability. In this study, five machine learning models: Random Forest (RF), K-Nearest-Neighbors (KNN), Adaptive-Boosting (AdaBoost), Light Gradient Boosting Machine (LightGBM), and Extreme-Gradient-Boosting (XGBoost) were developed and optimized using Grid Search to predict the CS of concrete containing glass powder (GP) and glass sand (GS). A dataset of 270 experimental samples was utilized, incorporating eight input parameters, including curing duration, cement content, GP, GS, water, density, sand, and basalt. Among the models, LightGBM demonstrated superior predictive performance during testing, achieving a determination coefficient (R² = 0.964) and Root-Mean-Square-Error (RMSE = 2.05 MPa), followed by XGBoost (R² = 0.955) and RF (R² = 0.953). In contrast, KNN and AdaBoost exhibited comparatively lower performance. SHAP and Partial Dependence Plot (PDP) analyses identified curing duration and cement content as the most influential parameters, while water and GP exhibited negative effects on CS. To enhance practical applicability, the LightGBM model was deployed through a user-friendly GUI, enabling rapid and reliable prediction of CS and providing an accurate, interpretable, and practical decision-support tool for sustainable concrete mixture design.

## Introduction

### Research background

The global construction industry is currently confronted with increasing pressure to minimize its environmental impact while simultaneously addressing the growing demand for infrastructure development. Cement production, a fundamental component of concrete, contributes approximately 7–8% of global anthropogenic carbon dioxide emissions, while the continuous extraction of natural aggregates leads to the depletion of finite geological resources^1,2^. In parallel, the management of post-consumer waste glass (WG) remains a persistent environmental concern, as millions of tons are disposed of in landfills annually despite its high recyclability and latent pozzolanic potential. These converging environmental challenges have accelerated the transition toward sustainable concrete technologies that utilize waste materials as functional constituents, thereby reducing environmental burdens while maintaining or enhancing structural performance^3^.

Among these alternatives, waste glass—when processed into glass powder (GP) or glass sand (GS)—has emerged as a promising substitute for conventional cementitious materials and natural aggregates. Numerous experimental studies have demonstrated that the partial replacement of cement with finely ground glass powder can enhance compressive strength, reduce permeability, and mitigate harmful alkali–silica reactions when used within optimal replacement ranges^4,5^. These improvements are primarily attributed to the high amorphous silica content of glass, which participates in pozzolanic reactions with calcium hydroxide to produce secondary calcium–silicate–hydrate (C–S–H) gel. This process contributes to densification of the interfacial transition zone and refinement of the pore structure, ultimately improving the durability and mechanical performance of concrete^6,7^. However, the influence of glass incorporation on concrete properties is inherently nonlinear and depends on multiple interacting factors, including particle size distribution, curing conditions, chemical admixtures, and synergistic effects with other supplementary cementitious materials. However, in terms of environmental performance, life cycle assessment studies consistently show that using WG as both powder and granular aggregate in concrete reduces global warming potential compared with conventional mixes by lowering clinker demand and diverting glass from landfills. Moreover, replacing a portion of cement and natural aggregates with recycled glass waste contributes to CO₂ emission reduction on a cradle‑to‑grave basis, supporting more sustainable, circular concrete with demonstrably lower carbon footprints and climate impacts^8,9^.

Conventional approaches to concrete mix design optimization are largely based on iterative experimental procedures, which are both time-consuming and resource-intensive. Although empirical regression models offer a simplified means of prediction, they often lack the capacity to capture the complex, multivariate interactions that govern concrete behavior, particularly in systems incorporating unconventional materials such as WG^10,11^. In recent years, the emergence of machine learning (ML) techniques has provided a transformative framework for modeling such complex systems. These data-driven approaches are capable of identifying intricate nonlinear relationships within experimental datasets, thereby enabling more accurate and efficient prediction of concrete properties^12–14^.

### Related work

The incorporation of WG into cementitious materials has attracted considerable attention as an effective strategy for reducing environmental impacts while improving concrete performance. Finely ground waste glass powder (WGP), composed primarily of amorphous silica (70–74% SiO₂), exhibits significant pozzolanic activity when its particle size is below approximately 75 μm, allowing it to react with calcium hydroxide and form additional C–S–H gel^15,16^. Numerous studies have demonstrated that replacing 10–20% of cement with WGP enhances compressive strength through improved particle packing and pozzolanic reactions^17–20^. However, higher replacement levels generally reduce strength because of cement dilution and lower hydration rates, while coarse glass particles may promote alkali–silica reaction (ASR) owing to their limited pozzolanic reactivity^21–24^. Likewise, waste glass can be utilized as GS to partially replace natural fine aggregates, where moderate replacement levels have been shown to maintain satisfactory mechanical performance while simultaneously reducing the environmental footprint of concrete^25–28^.

In recent years, the emergence of machine learning (ML) techniques has provided a transformative framework for modeling such complex systems. These data-driven approaches are capable of identifying intricate nonlinear relationships within experimental datasets, thereby enabling more accurate and efficient prediction of concrete properties^12–14^. Despite these advances, many high-performing ML models remain “black boxes,” providing limited insight into the physical influence of input variables on model predictions. Consequently, Explainable Artificial Intelligence (XAI) techniques, particularly SHapley Additive exPlanations (SHAP) and Partial Dependence Plots (PDPs), have emerged as powerful tools for interpreting feature importance and revealing the relationships between mixture parameters and predicted concrete properties.

On the other hand, life cycle assessment studies have shown that replacing one ton of cement with WGP can reduce carbon dioxide emissions by approximately 0.8–0.9 tons, even when accounting for the energy required for glass processing and grinding^29,30^. When coupled with the reduction in landfill disposal and the conservation of natural aggregate resources, the integration of waste glass into concrete aligns strongly with circular economy principles and offers a viable pathway toward more sustainable construction practices.

### Soft computing application

The application of XAI techniques for predicting concrete properties has expanded significantly over the past two decades, driven by advances in algorithms and the increasing availability of experimental datasets. Early studies primarily focused on artificial neural networks (ANNs), which demonstrated superior capability in capturing the nonlinear relationships between mix proportions and compressive strength compared to traditional empirical models. Yeh^10^ pioneered this field using a multilayer perceptron trained on a large dataset, achieving substantially lower prediction errors than conventional formulations. Subsequent research further improved ANN performance through optimization of network architecture and training strategies, establishing ANNs as a foundational approach in concrete strength prediction^31–33^. In recent years, the scope of ML applications in concrete modeling has broadened to include support vector machines (SVM), Random Forests (RF), and gradient boosting methods has shown strong performance, particularly for small to medium-sized datasets, due to their effective handling of nonlinear feature spaces^12,34^. Ensemble-based approaches, especially RF models, have gained prominence for their robustness and interpretability, often outperforming standalone ANN and SVM models^35,36^. More recently, Extreme Gradient Boosting (XGBoost) and Categorical Boosting (CatBoost) models have emerged as powerful techniques, offering enhanced predictive accuracy and reduced overfitting through regularization and efficient computation^14,37^.

To further improve model performance, hybrid approaches incorporating metaheuristic optimization techniques—such as particle swarm optimization (PSO) and Bayesian Optimization (BO) —have been employed for automated hyperparameter tuning^38,39^. Although deep learning models have also been explored, their effectiveness in concrete applications remains limited by dataset size and computational complexity^40,41^. A key advancement in recent ML-based studies is the emphasis on model interpretability. Techniques such as Shapley Additive Explanations (SHAP) enable detailed analysis of feature contributions, providing both global and local insights into model predictions^42^. The integration of interpretability methods has enhanced the reliability and transparency of ML models in concrete research, facilitating the identification of physically meaningful relationships beyond traditional statistical analyses^43^.

### Research gap and motivation

The integration of waste glass concrete with ML prediction remains a relatively recent yet rapidly evolving research area. Existing studies—primarily published after 2019—demonstrate strong predictive performance, with reported coefficients of determination (R²) typically ranging from 0.90 to 0.98 and root mean square errors below 4–6 MPa for compressive strength (CS) prediction^44–46^. Ensemble methods, particularly RF and gradient boosting, often achieve superior accuracy when combined with advanced hyperparameter optimization^47^. Key input parameters such as water-to-binder ratio, cement content, curing age, and glass replacement ratio have consistently been identified as the most influential features governing strength behavior^48,49^.

Despite these advancements, several critical limitations persist. Thus, this study proposes a comprehensive ML framework for predicting the CS of sustainable concrete incorporating glass powder and glass sand. The main contributions of this work are summarized as follows: (i) development and benchmarking of five advanced ML models to capture nonlinear relationships in CS prediction; (ii) systematic hyperparameter optimization to enhance model performance; (iii) rigorous evaluation using a multi-metric assessment framework; (iv) improved model interpretability through global and local explainability techniques, revealing key feature contributions and interactions; and (v) practical deployment of the best-performing model via a user-friendly GUI, enabling rapid prediction and supporting decision-making in engineering applications.

## Methodology

**Fig. 1:**
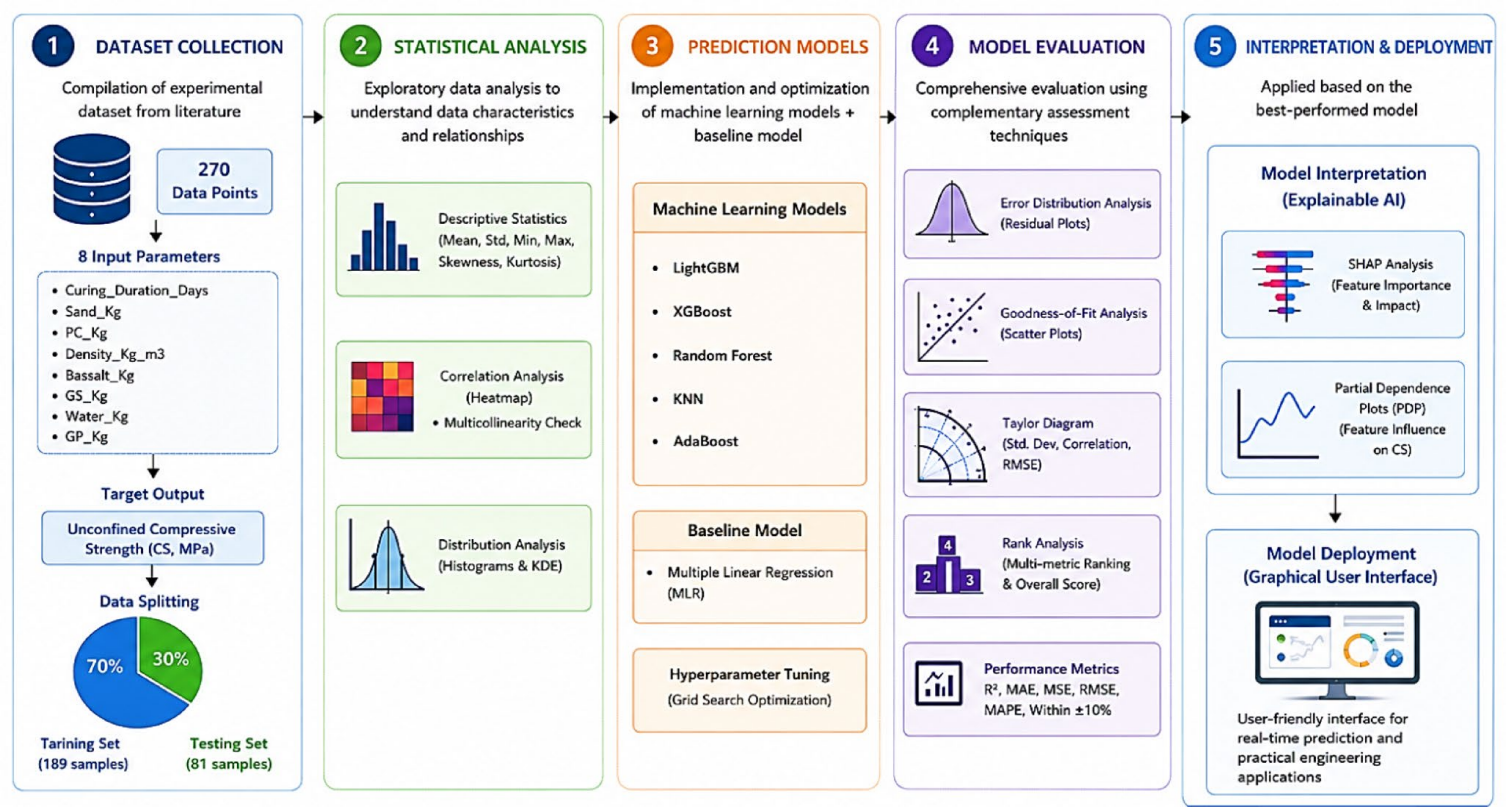
Methodological flowchart.

Figure 1 illustrates the structured methodological framework adopted in this study, presenting a comprehensive five-stage pipeline for predicting the CS of sustainable concrete incorporating glass powder and glass sand. The workflow begins with the dataset collection stage, where a total of 270 data points were compiled, comprising eight input parameters. The dataset was subsequently divided into training (70%) and testing (30%) subsets to ensure robust model development and validation. The second stage focuses on statistical analysis, including descriptive statistics, correlation heatmaps, and distribution visualization, to explore the dataset characteristics, identify potential multicollinearity, and understand input–output relationships. This step ensures data quality and provides essential insights for effective model training. In the Prediction Models phase, five state-of-the-art machine learning algorithms were implemented and optimized by grid search optimization; in addition, Multiple Linear Regression was incorporated as a baseline statistical model. These methods are well-suited for handling nonlinear relationships and complex interactions among features. The fourth stage involves model evaluation, where multiple complementary assessment techniques were employed, including analysis of error distribution, goodness via scatter plots, Taylor diagram, and rank analysis. These methods collectively provide a comprehensive evaluation of model accuracy and stability. In the fifth stage, the best predictive model was identified and further analyzed using explainable AI techniques, specifically SHAP and Partial Dependence Plots (PDP), to interpret feature importance and understand the influence of input variables on predictions. Finally, the developed framework is extended toward model deployment, where the optimal model is integrated into a user-friendly graphical interface, enabling practical application in real-time engineering scenarios. This end-to-end pipeline not only ensures high predictive accuracy but also enhances model transparency, interpretability, and usability for civil and structural engineering applications.

### Database collection

The dataset used in this study was compiled from the experimental investigation conducted by Shams et al.^50^, which evaluated the mechanical performance of sustainable concrete incorporating waste glass (WG) materials. The experimental program consisted of 30 unique concrete mixtures, in which glass powder (GP) was used as a partial replacement for Portland cement (PC), while glass sand (GS) partially replaced natural fine aggregate. Each mixture was tested at three curing ages (7, 28, and 56 days), with three replicate specimens per curing age, resulting in a total of 270 compressive strength measurements. Accordingly, the dataset comprises 270 observations derived from 30 distinct mixture designs. The complete mix proportions are provided in the Appendix.

Table 1 summarizes the descriptive statistics of all input variables considered in this study. The mixture constituents exhibit broad ranges, reflecting substantial variability in cement, glass powder, glass sand, aggregate contents (Sand and Basalt), water content, and curing duration. The skewness values indicate that most variables are approximately symmetric or only moderately skewed, suggesting that the dataset is well distributed without severe imbalance. Such variability is advantageous for machine learning because it enables the developed models to learn relationships over a wide range of mixture compositions, thereby improving their robustness and generalization capability.

To avoid information leakage arising from specimens originating from the same concrete mixture, the dataset was partitioned at the mixture level rather than at the individual specimen level. Specifically, 21 mixtures (70%) were assigned to the training set and the remaining 9 mixtures (30%) were reserved for independent testing. Consequently, all specimens corresponding to a given mixture, regardless of curing age or replicate, were kept exclusively within either the training or testing subset. This strategy provides a more rigorous assessment of model performance by preventing highly correlated samples from appearing in both subsets.

**Table 1:** Summary statistics for the data gathered.

Statistics → Variable ↓	Unit	Mean	Std.	Min	Max	25%	75%	Skewness
PC	kg/m^3^	352.00	38.23	280.0	400.0	320.0	380.0	-0.63
GP	kg/m^3^	48.00	38.23	0.0	120.0	20.0	80.0	0.63
GS	kg/m^3^	40.53	69.00	0.0	204.4	0.0	68.2	1.43
Density	kg/m^3^	2555.17	79.48	2352.0	2650.0	2516.0	2618.0	-1.05
Sand	kg/m^3^	626.90	65.46	463.9	684.0	614.1	665.8	-1.39
Basalt	kg/m^3^	1334.78	27.44	1290.7	1367.0	1320.7	1362.9	-0.24
Water	kg/m^3^	158.67	14.75	140.0	176.0	140.0	176.0	-0.14
Curing duration	Days	30.33	20.11	7	56	7	56	0.17

### Exploratory data analysis

#### Correlation analysis

Understanding the interdependencies between input and output features is crucial for finding the best prediction model, and this can be achieved by analyzing the correlation between variables. The Pearsosn correlation coefficient (*r*) is the most commonly used metric for this method, and it is essential in comprehending these relationships. It can be calculated as the covariance ratio (*cov*) of two variables to the product of their standard deviations^51^, as illustrated in Eq. (1).

1 $$r = \frac{{\mathrm{cov} \left( {a,b} \right)}}{{\sigma _{a} \sigma _{b} }} = \frac{{\sum\nolimits_{{i = 1}}^{n} {\left( {a_{i} - \bar{a}} \right)\left( {b_{i} - \bar{b}} \right)} }}{{\sqrt {\sum\nolimits_{{i = 1}}^{n} {\left( {a_{i} - \bar{a}} \right)^{2} } } \sqrt {\sum\nolimits_{{i = 1}}^{n} {\left( {b_{i} - \bar{b}} \right)^{2} } } }}$$

Where *n* is the number of a dataset, $\bar{a}$and $\bar{b}$ are the mean of two variables (*a*, *b*).

**Fig. 2:**
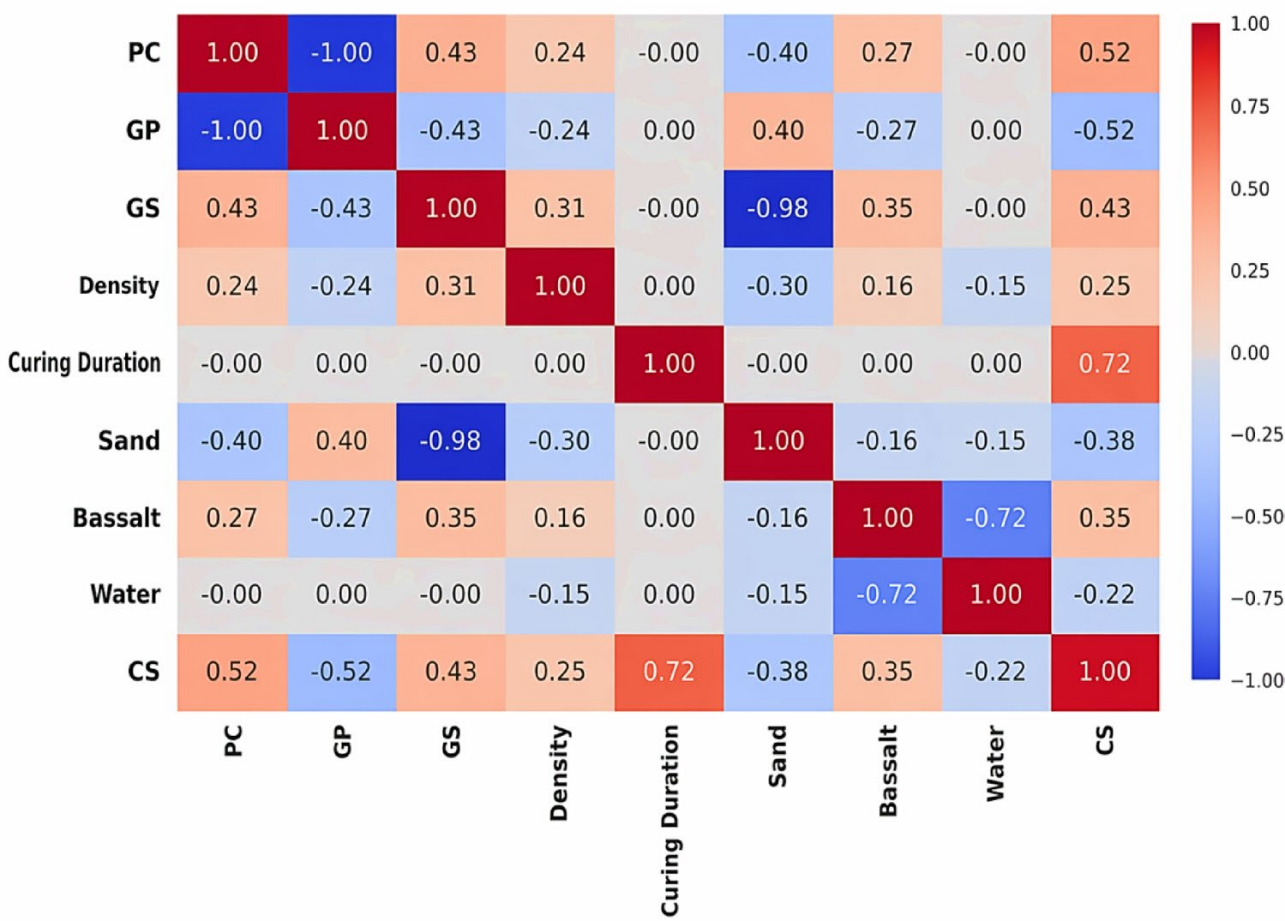
Correlation matrix for the employed variables.

Understanding the statistical interdependencies between variables is vital for building effective predictive models, particularly when working with multivariate datasets. Figure 2 presents a Pearson correlation heatmap that visually and quantitatively describes the strength and direction of linear relationships among the eight independent variables as well as the dependent variable (i.e., CS). A strong inverse correlation is observed between PC and GP with a coefficient of − 1.00, confirming that GP directly replaces cement in the mixture design. Similarly, a very strong negative correlation exists between GS and natural sand (− 0.98), reflecting the substitution mechanism of GS for fine aggregate. These strong correlations validate the consistency and logical structure of the experimental dataset.

In relation to the target variable, CS exhibits a strong positive correlation with curing duration (0.72), indicating that strength development is primarily governed by continued cement hydration and progressive microstructural densification, which enhance the formation of calcium silicate hydrate (C–S–H) gel and reduce pore connectivity, leading to higher compressive strength^52^. A moderate positive correlation is also observed between CS and Portland cement (PC) (0.52), reflecting the fundamental role of cement as the primary binding material responsible for strength development through hydration reactions. Similarly, glass sand (GS) shows a moderate positive correlation with CS (0.43), suggesting that replacing natural sand with appropriately graded glass sand can improve particle packing density and contribute to a denser concrete matrix, provided that the replacement level remains within an optimum range^53^.

Conversely, glass powder (GP) exhibits a moderate negative correlation with CS (− 0.52). This trend may be attributed to the partial replacement of cement by GP, which reduces the amount of reactive cement available for hydration. Although finely ground GP possesses pozzolanic activity, excessive replacement levels may delay strength development due to the dilution effect and slower pozzolanic reactions, particularly at early ages^54,55^. Likewise, water content shows a weak negative correlation with CS (− 0.22), which is consistent with the well-established influence of the water-to-cement ratio, where higher water contents increase capillary porosity and weaken the hardened cement matrix^56^. A moderate negative correlation is also observed between sand content and CS (− 0.38), suggesting that excessive fine aggregate may reduce the effectiveness of the cement paste in binding the aggregate skeleton and adversely affect internal packing characteristics.

Among the remaining variables, basalt (coarse aggregate) demonstrates a moderate positive correlation with CS (0.35), indicating that the high stiffness and mechanical strength of basalt aggregate can enhance load transfer and aggregate interlocking, thereby improving CS^57^. Furthermore, water content exhibits a strong negative correlation with basalt (− 0.72), reflecting the mixture proportioning strategy adopted to maintain workability under varying aggregate contents rather than a direct physical interaction. Finally, density shows relatively weak correlations with most variables, including CS (0.25), suggesting that although denser mixtures generally contain fewer internal voids, density alone is not the dominant factor governing CS compared with PC, curing duration, and replacement materials. The presence of both strong and moderate correlations among variables ensures that the dataset captures meaningful physical relationships, which is essential for developing accurate and interpretable ML models^58^.

#### Distribution characteristics

**Fig. 3:**
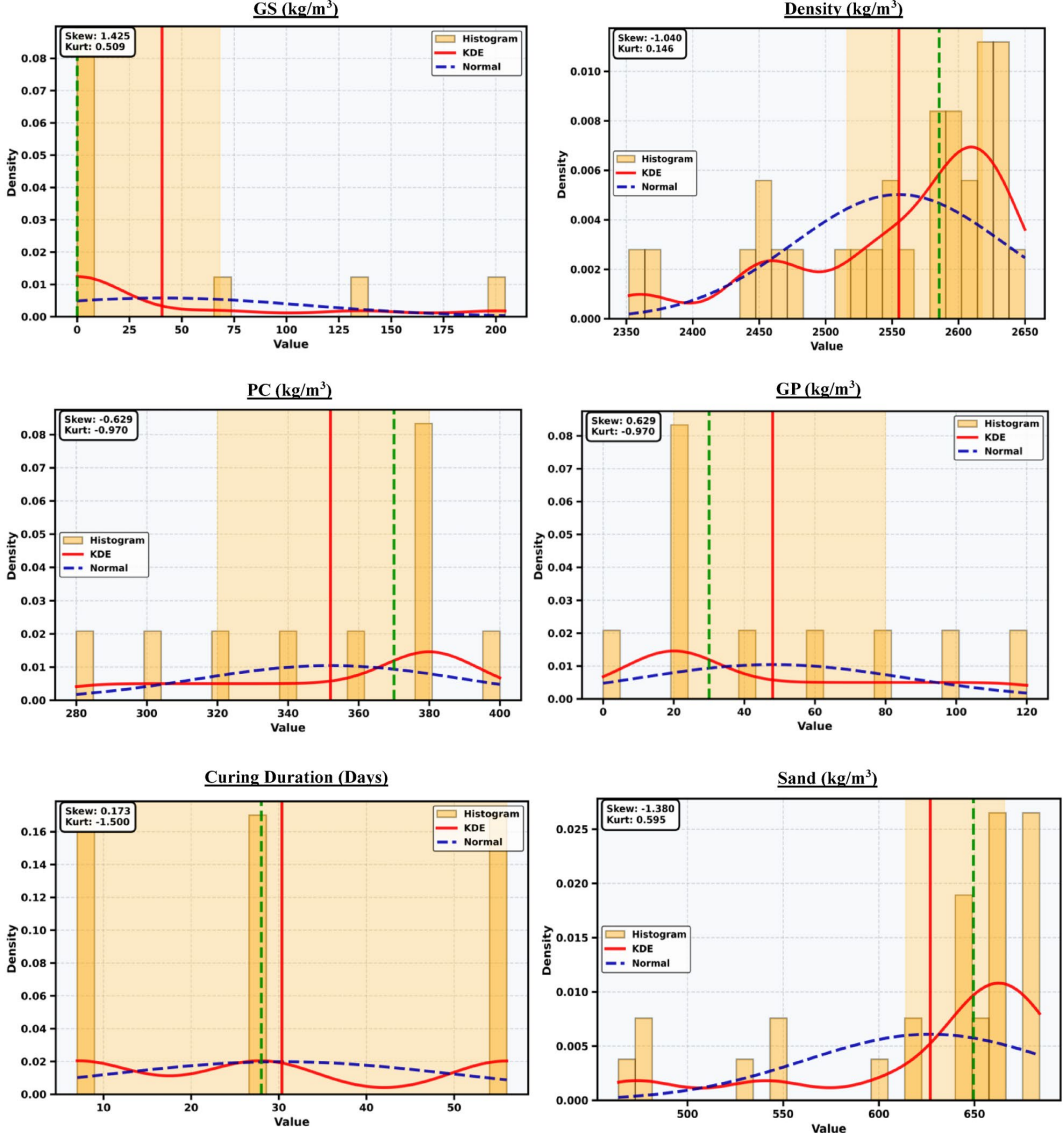

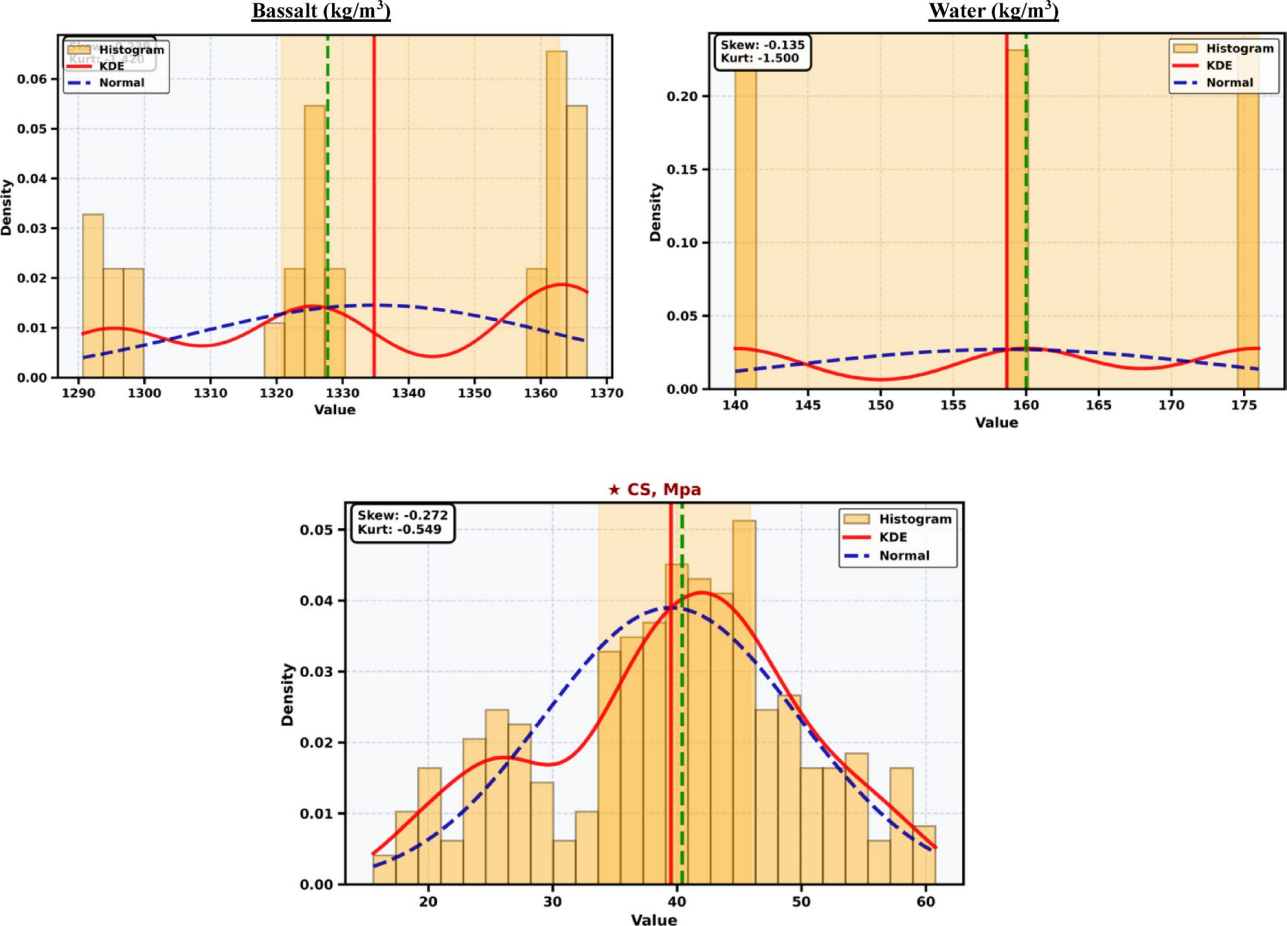
Statistical distribution of the input variables CS via histograms, KDE, and fitted normal distribution.

Figure 3 illustrates the statistical distribution of all input variables and the target CS using histograms overlaid with kernel density estimation (KDE) curves and the corresponding normal distribution. The figure provides a visual assessment of the distribution characteristics, including central tendency, dispersion, skewness, and kurtosis, which are important for evaluating the suitability of the dataset for ML applications. The distributions of the mixture constituents exhibit varying degrees of symmetry. PC and density show slight negative skewness (− 0.629 and − 1.040, respectively), indicating that most observations are concentrated toward the higher end of their respective ranges. Conversely, GP and GS exhibit positive skewness (0.629 and 1.425), reflecting the larger number of mixtures containing relatively low replacement levels with fewer mixtures incorporating high glass contents. These trends are consistent with practical concrete mix design, where high replacement ratios are generally less common because of their potential influence on mechanical performance.

The remaining input variables demonstrate relatively mild departures from normality. Water content and curing duration are nearly symmetric, with skewness values close to zero (− 0.135 and 0.173, respectively), while sand, basalt, and density exhibit moderate negative skewness, indicating a slightly greater concentration of observations at higher material contents. Moreover, the kurtosis values of all variables remain within a relatively narrow range, suggesting the absence of excessively peaked or heavy-tailed distributions. This indicates that the dataset is free from severe distributional abnormalities that could adversely affect the training stability of machine learning models.

The distribution of the target variable (CS) is approximately bell-shaped with a slight negative skewness (− 0.272) and a kurtosis of − 0.549, indicating a moderately flat (platykurtic) distribution compared with an ideal normal distribution. The close agreement between the histogram, KDE curve, and theoretical normal distribution demonstrates that compressive strength values are well distributed across the investigated range without excessive clustering at either extreme. Such a balanced distribution is desirable for supervised learning because it enables the models to learn representative relationships throughout the entire response domain rather than being biased toward a limited range of strength values. Overall, Fig. 3 demonstrates that the collected dataset covers a broad spectrum of mixture compositions while maintaining balanced statistical characteristics. Although several input variables exhibit moderate positive or negative skewness, none displays severe distributional imbalance or extreme outliers. These characteristics provide a diverse and representative learning space, which enhances the robustness and generalization capability of the developed ML models.

### Overview of adopted models

Machine learning (ML) algorithms are generally categorized into three main types: supervised, unsupervised, and semi-supervised learning^59^. In the present study, supervised learning algorithms were exclusively employed, as the target variable (i.e., CS) was clearly defined and available within the dataset. Supervised models utilize labeled data to establish relationships between input features and output responses, enabling accurate prediction and facilitating the identification of underlying patterns governing concrete behavior. The ML models adopted in this study, namely Random Forest (RF), K-Nearest Neighbors (KNN), Adaptive Boosting (AdaBoost), Light Gradient Boosting Machine (LightGBM), and Extreme Gradient Boosting (XGBoost), were selected to represent a diverse range of algorithmic paradigms, including bagging-based ensembles, instance-based learning, and boosting techniques. In addition, Multiple Linear Regression (MLR) was incorporated as a baseline statistical model to provide a benchmark against which the predictive improvements achieved by the advanced machine learning algorithms could be objectively evaluated^60^. This selection ensures a comprehensive and unbiased evaluation of predictive performance for sustainable concrete incorporating GP and GS. Ensemble-based models such as RF, LightGBM, and XGBoost are particularly effective in capturing the complex nonlinear and multivariate relationships inherent in concrete mixtures, while AdaBoost provides a comparatively simpler boosting framework for benchmarking purposes. In contrast, KNN serves as a baseline non-parametric model capable of modeling local data structures without assuming any predefined functional relationship^39^. A brief description of these models, along with their advantages and limitations, is presented in Table 2.

To ensure a fair and reliable comparison, the hyperparameters of all models were systematically optimized using Grid search (GS) optimization. For the MLR model, the parameters included feature normalization, second-order polynomial expansion, univariate feature selection, and unconstrained regression coefficients. For the RF model, key parameters including the number of trees (n_estimators), maximum tree depth (max_depth), and minimum samples required for splitting (min_samples_split) were tuned to balance model bias and variance. In boosting-based models (AdaBoost, LightGBM, and XGBoost), critical hyperparameters such as learning rate, number of estimators, and tree depth were optimized to enhance convergence and mitigate overfitting. Additionally, LightGBM-specific parameters (e.g., num_leaves and feature fraction) and XGBoost-specific parameters (e.g., subsample, colsample_bytree, and gamma) were carefully adjusted to improve generalization performance. For the KNN model, the number of neighbors (*k*), distance metric, and weighting scheme were optimized to ensure effective local approximation of the dataset. This systematic model selection and hyperparameter tuning strategy ensured that all algorithms operated under near-optimal conditions, thereby enabling a robust and meaningful comparison of their predictive capabilities. Finally, all analyses were performed using Python 3.12. The primary software packages included scikit-learn (v1.6.1) for data preprocessing, model development, hyperparameter optimization, and performance evaluation.

**Table 2:** Overview of implemented models in current study.

Model	Model type	Description	Advantages	Limitations	Reference
MLR	Statistical Regression	The relationship between the target variable and multiple input variables by fitting a linear equation using the least squares method.	- Simple, interpretable, and computationally efficient. - Provides a transparent baseline for comparing advanced machine learning models.	- Linear relationship between predictors and the target variable. - Limited ability to capture complex nonlinear interactions among variables.	^61^
RF	Ensemble (Bagging)	RF constructs multiple decision trees using bootstrapped samples and aggregates their predictions to improve accuracy and stability.	- High predictive accuracy and robustness to noise and overfitting. - Effectively captures nonlinear relationships and handles mixed-type data.	- Computationally expensive for large numbers of deep trees. - Limited interpretability compared to single decision trees.	^62^
KNN	Instance-Based Learning	KNN predicts outcomes based on the similarity of input data to its nearest neighbors in feature space.	- Simple, non-parametric, and assumption-free. - Capable of capturing local data patterns effectively.	- Sensitive to feature scaling and irrelevant variables. - High computational cost and memory usage for large datasets.	^63^
AdaBoost	Ensemble (Boosting)	AdaBoost sequentially trains weak learners, emphasizing misclassified or high-error samples in subsequent iterations.	- Enhances the performance of weak learners through iterative refinement. - Simple and effective for structured datasets.	- Sensitive to noise and outliers. - Less competitive than advanced boosting methods.	^64^
LightGBM	Ensemble (Boosting)	LightGBM is a gradient boosting framework that uses leaf-wise tree growth and histogram-based splitting for efficient computation.	- High computational efficiency and low memory usage. - Achieves competitive or superior accuracy with faster training.	- Prone to overfitting if not properly tuned. - Requires careful parameter selection.	^65^
XGBoost	Ensemble (Boosting)	XGBoost is an optimized gradient boosting algorithm incorporating regularization and second-order optimization for enhanced performance.	- High accuracy with strong regularization to reduce overfitting. - Scalable and effective for complex nonlinear problems.	- Computationally intensive compared to simpler models. - Sensitive to hyperparameter tuning and noisy data.	^66^

### Performance assessment

Assessing model performance is essential to ensure the scientific reliability and practical applicability of predictive models^67^. In ML techniques, the training dataset is primarily used to develop the model and evaluate its ability to learn patterns from observed data, whereas the testing dataset plays a critical role in assessing the model’s generalization capability on unseen data. This distinction is fundamental to avoid overfitting and to ensure that the developed models can reliably predict new instances beyond the training domain. In the present study, model evaluation is conducted using both visual and quantitative approaches. Visual assessments, through graphical representations, provide intuitive insights into prediction trends, deviations, and potential anomalies that may not be evident from numerical metrics alone. These plots facilitate a deeper understanding of model behavior and consistency across different data ranges.

In addition to visual analysis, quantitative evaluation metrics are employed to provide an objective and standardized assessment of predictive performance. A set of five widely used regression metrics—Determination Coefficient (R²), Mean Square Error (MSE), Root Mean Square Error (RMSE), Mean Absolute Error (MAE), and Mean Absolute Percentage Error (MAPE)—are utilized to comprehensively evaluate model accuracy and reliability. The R² value indicates the degree of agreement between predicted and actual values, with values approaching 1.0 representing superior model performance. Conversely, MSE, RMSE, MAE, and MAPE quantify prediction errors, where values closer to zero indicate higher accuracy and lower deviation. The combined use of these metrics provides a robust evaluation framework, enabling meaningful comparison between different models and ensuring the selection of the most reliable predictive approach. The mathematical formulations and ideal ranges of these performance indicators are presented in Table 3.

**Table 3:** Performance metrics to assess the developed ML models.

Regression metric	Equation	Ideal value
Determination coefficient	$\:{\mathrm{R}}^{2}=1-\frac{{\sum\:}_{i=1}^{n}{\left({y}_{i}-\widehat{{y}_{i}}\right)}^{2}}{{\sum\:}_{i=1}^{n}{\left({y}_{i\:}-\stackrel{-}{y}\right)}^{2}}$	1
Mean squared error	$\:\mathrm{M}\mathrm{S}\mathrm{E}=\frac{\sum\:_{i=1}^{\:n}{\left({y}_{i}-\widehat{{y}_{i}}\right)}^{2}}{n}$	0
Root mean squared error	$\:\mathrm{R}\mathrm{M}\mathrm{S}\mathrm{E}=\sqrt{\frac{\sum\:_{i=1}^{\:n}{\left({y}_{i}-\widehat{{y}_{i}}\right)}^{2}}{n}\:}$	0
Mean absolute error	$\:\mathrm{M}\mathrm{A}\mathrm{E}=\frac{\sum\:_{i=1}^{\:n}\left|{y}_{i}-\widehat{{y}_{i}}\right|}{n}$	0
Mean absolute percentage error	$\:\mathrm{M}\mathrm{A}\mathrm{P}\mathrm{E}=\frac{\sum\:\left|\frac{{y}_{i}-\widehat{{y}_{i}}}{{y}_{i}}\right|}{n}$	0

Where $\:{y}_{i}$ and $\:\widehat{{y}_{i}}\:$are actual and forecasted *i*^th^ values, respectively and $\overline{{\hat{y}}}$is the average of forecasted values.

### Evaluation of the studied features

The interpretation and transparency of ML models are essential for ensuring their reliability, trustworthiness, and practical utility. Feature sensitivity analysis offers essential insights regarding the significance of input features in shaping model predictions. The prominent method employed for feature sensitivity analysis is SHapley-Additive-exPlanation (SHAP). This method elucidates the decision-making processes of ML models, offering researchers and practitioners a deeper understanding of feature contributions.

SHAP is an advanced method grounded in the principles of cooperative game theory, aimed at interpreting and assessing machine learning models at both global and detailed levels^68^. SHAP quantifies the contribution of each input feature to the prediction process by calculating Shapley values, thereby effectively capturing the intricate interactions between features and model outputs. SHAP serves as a prominent approach in Explainable Artificial Intelligence (XAI), offering insights into the influence of features on model predictions and emphasizing the significance of each variable.

## Results and insights

### Hyperparameter optimization of ML models

Prior to model training, all input features were standardized using the StandardScaler algorithm within a scikit-learn Pipeline. The scaler was fitted exclusively on the training data during each cross-validation (CV) iteration, and the same transformation was subsequently applied to the corresponding validation and independent testing data. Integrating feature standardization and model training within a unified pipeline prevents data leakage and ensures an unbiased evaluation of model performance. Therefore, the predictive models were optimized by systematically tuning their hyperparameters using Grid Search (GS) combined with cross-validation. The optimal hyperparameter settings obtained for each model are summarized in Table 4, together with a brief description of the role of each parameter in controlling the learning process, model complexity, and prediction performance.

As shown in Table 4, the optimal hyperparameters differed according to the underlying learning mechanism of each algorithm. For the polynomial MLR model, the best performance was achieved using Min-Max-Scaler for feature normalization, a second-order polynomial expansion (degree = 2), and univariate F-regression retaining the top 15 features, while fit_intercept and positive_coefficients were both set to False. The optimized RF model employed 200 decision trees, bootstrap sampling, a maximum tree depth of 10, and minimum values of 2.0 for both samples per split and samples per leaf, providing an effective balance between prediction accuracy and model complexity. For the KNN model, the optimal configuration consisted of five nearest neighbors (*k* = 5) using the Euclidean distance metric (*p* = 2) with distance weighting, enabling more reliable prediction based on local neighborhood information.

For the boosting algorithms, the optimization process primarily focused on controlling the learning rate, tree complexity, and ensemble size. Both AdaBoost, LightGBM, and XGBoost achieved optimal performance with a learning rate of 0.1, indicating that gradual learning produced better generalization than more aggressive updates. AdaBoost further employed the square loss function with 200 weak learners, whereas LightGBM was optimized using a maximum tree depth of 3, 100 boosting iterations, 31 leaves, and a minimum of 10 samples per leaf to efficiently capture nonlinear relationships while limiting overfitting. Similarly, the optimal XGBoost model utilized a maximum tree depth of 3, 100 boosting trees, and row and column subsampling ratios of 0.8, thereby increasing model diversity and enhancing generalization performance. Collectively, these optimized hyperparameter settings enabled each algorithm to achieve a suitable balance between learning efficiency, predictive accuracy, and robustness, and were subsequently adopted for the development of the final predictive models.

**Table 4:** Optimized hyperparameters of models obtained via cross-validated GS.

Model	Hyperparameter	Optimal value	Description
MLR (Polynomial)	Scaler	Min-Max-Scaler	Normalises all features to [0, 1] before polynomial expansion
	Polynomial Degree	2	Degree of polynomial feature expansion applied to inputs
	Interaction Only	False	Includes both squared terms and cross-product interaction features
	Feature Selector	f_regression; k = 15	Univariate F-statistic selector retaining the top 15 features
	Fit Intercept	False	Intercept term suppressed; data normalized before fitting
	Positive Coefficients	False	Regression coefficients are unconstrained in sign
RF	Bootstrap	True	Bootstrapped sampling of training data applied for each tree
	Max. Depth	10	Maximum depth permitted for individual decision trees
	Min. Samples per Leaf	2	Minimum number of samples required at a terminal (leaf) node
	Min. Samples to Split	2	Minimum samples required to split an internal node
	No. of Estimators	200	Total number of trees constructed in the forest
KNN	Algorithm	Auto	Selects the most appropriate algorithm automatically
	No. of Neighbors (*k*)	5	Number of nearest neighbors considered for prediction
	Distance Metric (*p*)	2 (Euclidean)	Minkowski metric with *p* = 2 corresponds to Euclidean distance
	Weight Function	Distance	Closer neighbors receive proportionally higher weights
AdaBoost	Learning Rate	0.1	Shrinkage factor applied to each weak learner’s contribution
	Loss Function	Square	Squared-error loss used for updating sample weights
	No. of Estimators	200	Total number of sequential weak learners (decision stumps)
LightGBM	Learning Rate	0.1	Step-size shrinkage applied during gradient boosting
	Max. Depth	3	Maximum tree depth; controls overall model complexity
	Min. Data in Leaf	10	Minimum number of samples required in each leaf node
	No. of Estimators	100	Total number of boosting iterations performed
	No. of Leaves	31	Maximum number of leaves allowed per tree
XGBoost	Column Subsample	0.8	Fraction of features randomly sampled per tree construction
	Learning Rate	0.1	Step-size shrinkage applied to prevent overfitting
	Max. Depth	3	Maximum depth of each gradient-boosted tree
	No. of Estimators	100	Total number of gradient-boosted trees
	Row Subsample	0.8	Fraction of training samples used per boosting round

### Visual assessment of prediction models

#### Error analysis

**Fig. 4:**
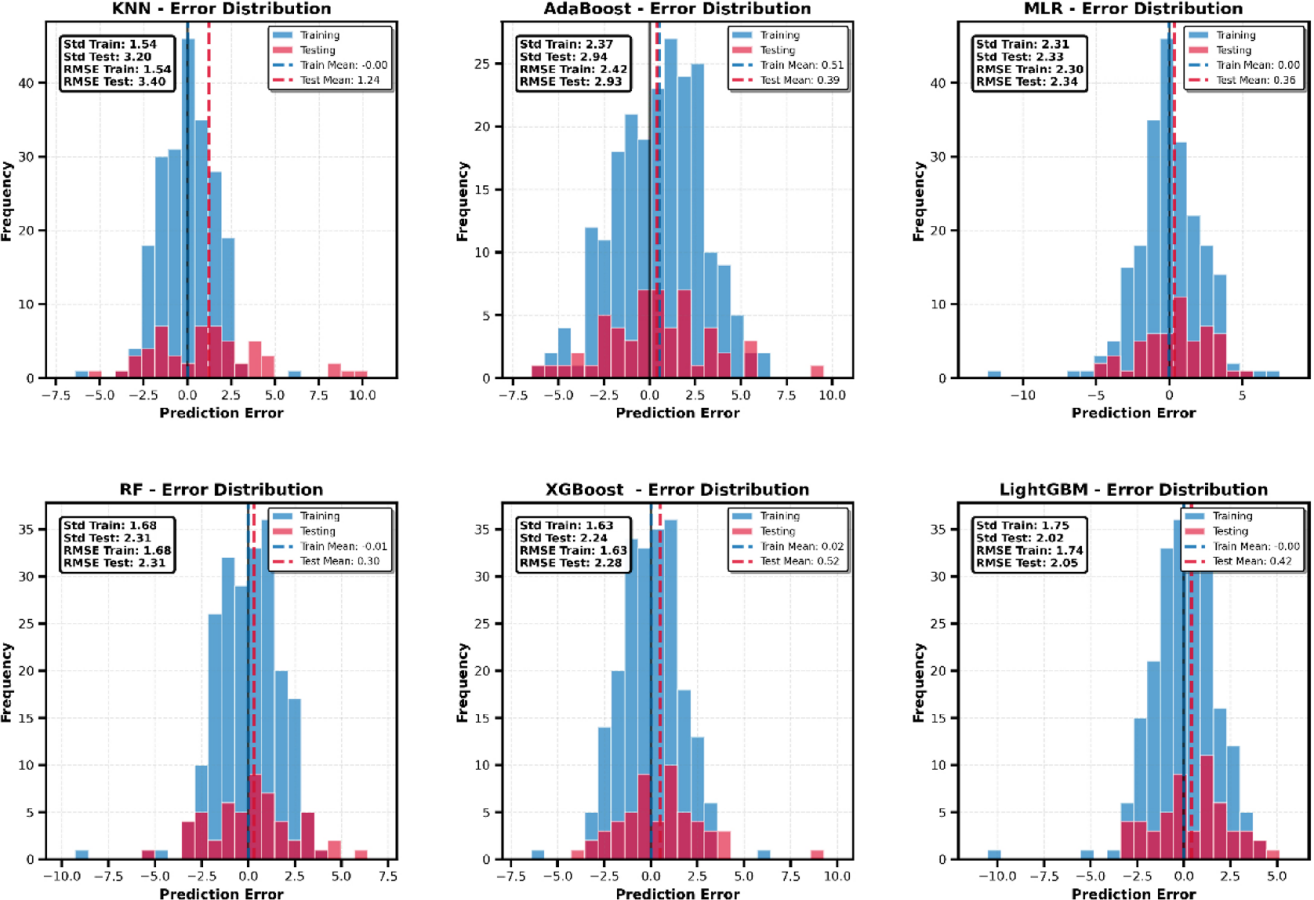
Error distribution analysis during the training and testing stages for all employed models.

Figure 4 presents the error distributions of the six predictive models for both the training and testing datasets. The histograms illustrate the frequency of prediction errors, while the dashed vertical lines represent the mean error for each dataset. In addition, the standard deviation (Std) and RMSE are reported to quantify the dispersion and magnitude of prediction errors. An ideal predictive model should exhibit a narrow error distribution centered around zero, indicating low prediction bias and high prediction accuracy. Therefore, comparison of the error distributions provides valuable insight into the robustness and generalization capability of the developed machine learning models beyond conventional performance metrics.

Among the ML models, KNN exhibited the widest prediction error distribution. The testing RMSE increased to 3.40 MPa, compared with 1.54 MPa during training, while the standard deviation increased from 1.54 MPa to 3.20 MPa. Furthermore, the testing mean error reached 1.24 MPa, indicating a noticeable positive prediction bias. The large discrepancy between training and testing performance suggests that the local neighborhood relationships learned by KNN were less transferable to unseen data. The AdaBoost model demonstrated a substantial improvement over KNN. The training and testing RMSE values were 2.42 MPa and 2.93 MPa, respectively, with corresponding standard deviations of 2.37 MPa and 2.94 MPa. The prediction errors remained reasonably centered around zero, with mean errors of 0.51 MPa for training and 0.39 MPa for testing, indicating relatively low systematic bias. The polynomial MLR model yielded a relatively low prediction error, with RMSE values of 2.30 MPa for the training set and 2.34 MPa for the testing set, together with standard deviations of 2.31 MPa and 2.33 MPa, respectively. Notably, its predictive performance was comparable to, and slightly superior to, that of the AdaBoost and KNN models on the testing dataset, highlighting that an appropriately configured polynomial regression model can provide a strong baseline despite its lower complexity.

The three tree-based ensemble models (i.e., RF, XGBoost, and LightGBM) produced the narrowest and most symmetric error distributions, demonstrating their superior predictive capability. Among them, LightGBM achieved the lowest testing error, with an RMSE of 2.05 MPa and a testing standard deviation of 2.02 MPa, compared with 1.74 MPa for both training RMSE and standard deviation. Similarly, XGBoost yielded a testing RMSE of 2.28 MPa with a testing standard deviation of 2.24 MPa, while maintaining a very small training RMSE of 1.63 MPa. The RF model also demonstrated excellent performance, recording testing and training RMSE values of 2.31 MPa and 1.68 MPa, respectively. These comparatively narrow error distributions indicate that the ensemble tree models effectively captured the nonlinear relationships between the mixture variables and CS. Overall, LightGBM achieved the narrowest testing error distribution and the lowest testing RMSE, followed closely by XGBoost and RF. Although MLR did not outperform the tree-based ensemble models, it demonstrated predictive performance comparable to AdaBoost and KNN, confirming its suitability as a reliable baseline model.

#### Goodness of fit

**Fig. 5:**
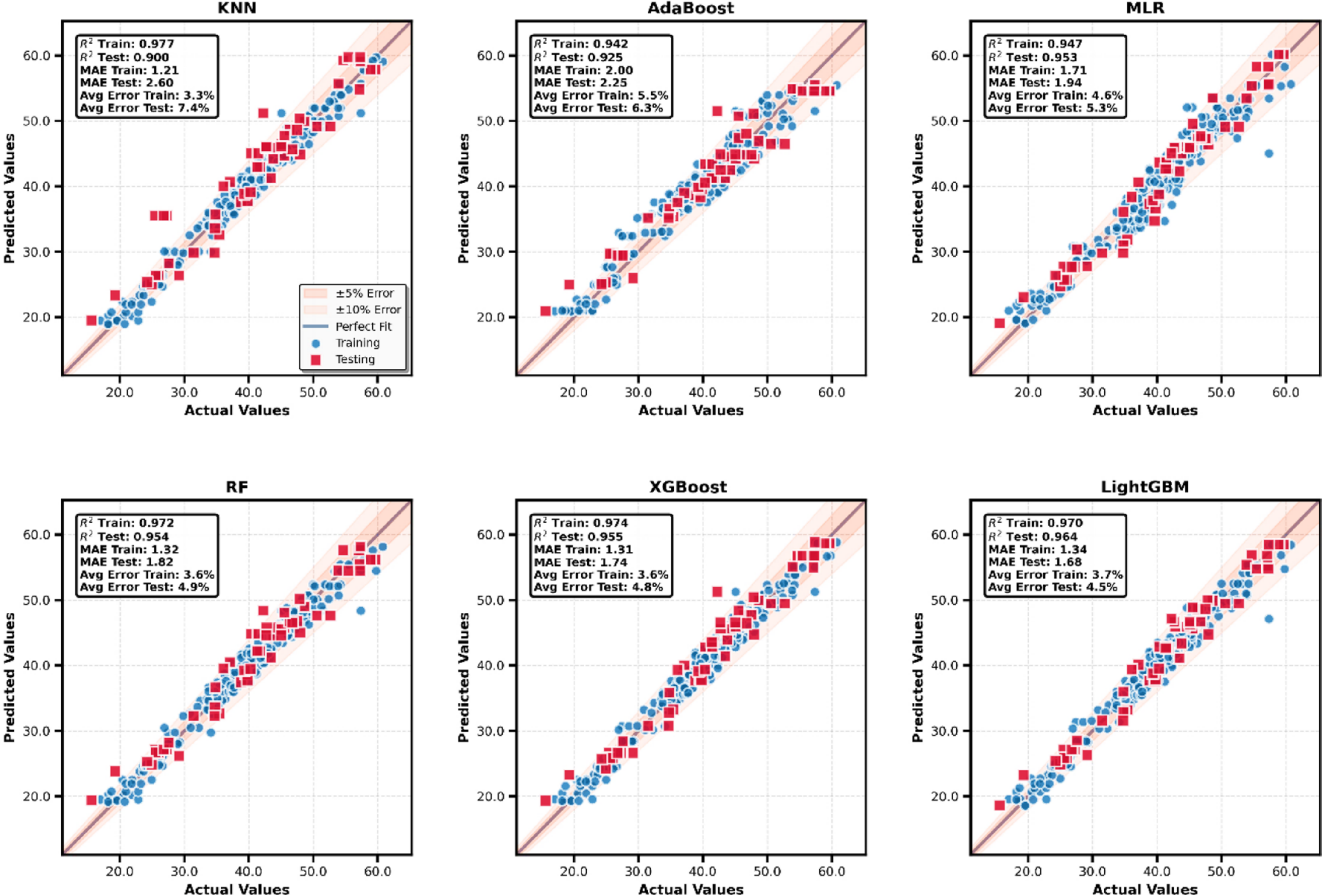
Actual versus predicted CS for each model during training and testing stages with ± 5 and ± 10% error bands.

Figure 5 compares the predictive performance of the six developed models using scatter plots of the predicted versus measured CS values for both the training and testing datasets. The solid diagonal line represents the ideal one-to-one relationship, while the shaded regions indicate prediction errors within ± 5% and ± 10% of the measured values. A model exhibiting high predictive capability is characterized by data points tightly clustered around the one-to-one line, high (R²), low (MAE), and a large proportion of predictions falling within the predefined error bounds. These graphical comparisons provide valuable insight into both the accuracy and performance of the developed models.

For the training dataset, all six models demonstrated excellent agreement between the predicted and measured CS values, with R² values ranging from 0.942 to 0.977. Among them, KNN achieved the highest training accuracy (R² = 0.977, MAE = 1.21 MPa) and exhibited 95.83% of predictions within the ± 10% error band. The tree-based ensemble models also showed strong performance, with RF, XGBoost, and LightGBM producing training R² values of 0.972, 0.974, and 0.970, respectively, accompanied by relatively low MAE values ranging from 1.32 to 1.74 MPa. The polynomial MLR model also demonstrated competitive training performance, achieving R² = 0.947 with an MAE of 1.71 MPa, while 93.06% of its predictions were contained within the ± 10% error limits. Although AdaBoost exhibited the lowest training R² (0.942) and the highest training MAE (2.25 MPa), the overall agreement between predicted and experimental values remained satisfactory.

The comparison becomes more informative when evaluating the testing dataset, which reflects the generalization capability of the developed models. As illustrated in Fig. 5, all models maintained relatively high prediction accuracy, with testing R² values ranging from 0.900 to 0.964. LightGBM achieved the highest predictive performance, producing the largest testing coefficient of determination (R² = 0.964), the lowest testing MAE (1.68 MPa), and the highest percentage of predictions within the ± 10% error band (94.44%). XGBoost followed closely with R² = 0.955, MAE = 1.74 MPa, and 92.59% of predictions within the ± 10% interval, while RF achieved R² = 0.954, MAE = 1.82 MPa, and 90.74% of predictions within the same tolerance.

Interestingly, the polynomial MLR model demonstrated competitive predictive capability despite its simpler mathematical formulation. The model achieved a testing R² of 0.953 with a relatively low MAE of 1.94 MPa, while 88.89% of the predictions fell within the ± 10% error band. These results indicate that the polynomial feature expansion enabled MLR to capture a considerable proportion of the nonlinear relationships governing compressive strength, allowing it to perform comparably with several machine learning algorithms. In contrast, AdaBoost achieved a testing R² of 0.925 with an MAE of 2.20 MPa, whereas KNN produced the lowest testing performance (R² = 0.900 and MAE = 2.60 MPa) and the smallest proportion of predictions within the ± 10% interval (83.33%), suggesting comparatively lower generalization capability for unseen mixtures. The noticeable reduction in KNN performance from the training to the testing dataset indicates a high-variance learning behavior. This can be attributed to the relatively small number of neighbors (*k* = 5) combined with distance-based weighting, which increases the model’s sensitivity to local training samples. Consequently, KNN captures local data patterns effectively during training but exhibits reduced generalization capability when predicting previously unseen mixtures.

#### Taylor diagram

**Fig. 6:**
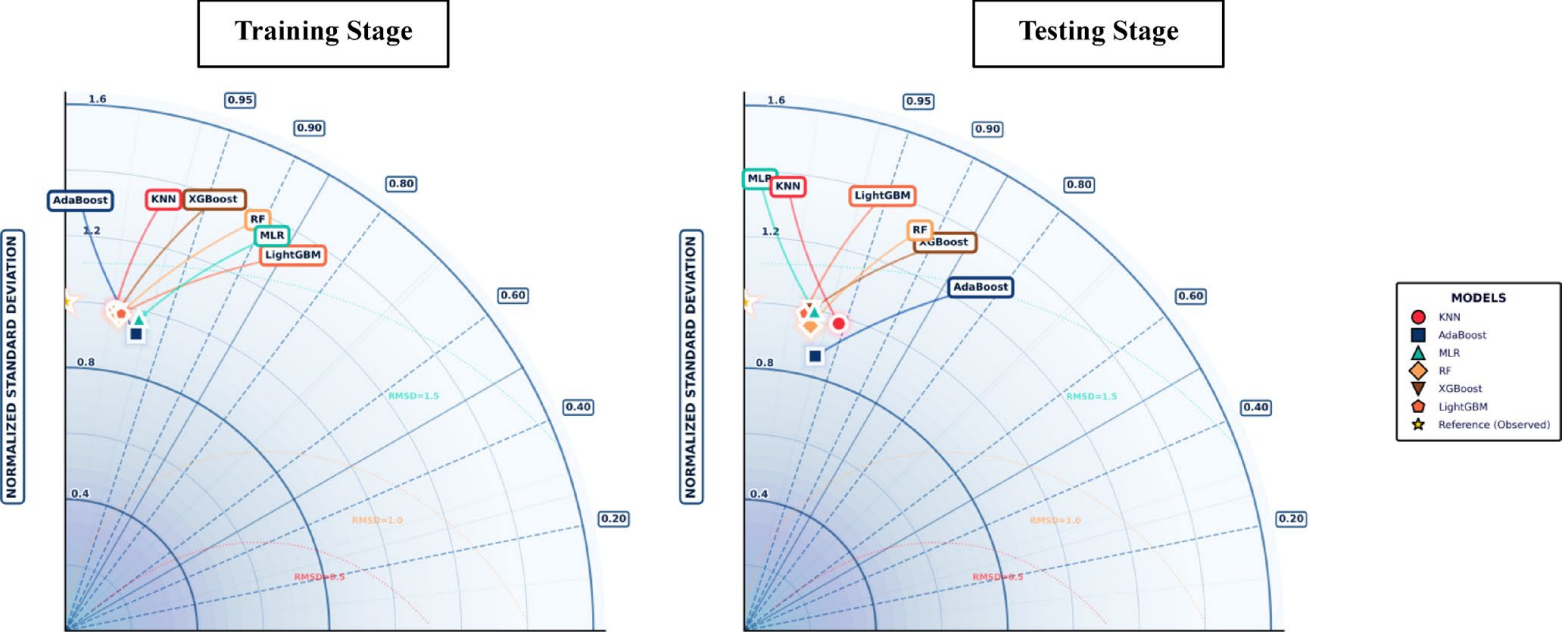
Evaluation of adopted models via Taylor diagrams during training and testing stages.

Figure 6 presents the Taylor diagrams for the training and testing datasets, providing a complementary assessment of model performance through the combined evaluation of normalized Std. (*σ*_actual_/*σ*_model_) and centered root mean square deviation (RMSD) relative to the experimental observations. Unlike the previous figures, which primarily quantify prediction accuracy using statistical performance indices, the Taylor diagram additionally evaluates each model’s ability to reproduce the variability of the observed compressive strength. Consequently, models positioned closer to the reference point simultaneously exhibit a normalized standard deviation approaching unity and a smaller RMSD, indicating better agreement with the statistical characteristics of the experimental dataset.

For the training stage, the polynomial MLR and LightGBM models are positioned closest to the reference normalized standard deviation (*σ*_actual_/*σ*_model_ ≈ 1.0), indicating that both models accurately reproduced the variability of the measured compressive strength. In comparison, RF and XGBoost exhibited slightly larger normalized standard deviations (approximately 1.12 ~ 1.14), while KNN and AdaBoost showed the greatest deviation from the reference value (≈ 1.15 and ≈ 1.24, respectively), suggesting a modest overestimation of data variability. Nevertheless, all models remained within a relatively narrow range of normalized standard deviations, demonstrating their capability to capture the overall statistical dispersion of the experimental observations.

A similar trend is observed for the testing stage, although clearer differences between the models become evident. LightGBM remains closest to the reference point, with a normalized standard deviation nearly identical to the observed data and the smallest centered RMSD, indicating excellent agreement with both the magnitude and variability of the experimental compressive strength. The MLR, RF, and XGBoost models also maintained normalized standard deviations close to unity (approximately 1.02 ~ 1.12) and exhibited similarly low RMSD values, confirming their ability to preserve the statistical characteristics of the testing dataset. These results indicate that the polynomial feature expansion substantially improved the capability of the MLR model to reproduce the observed variance, yielding statistical behavior comparable to that of the ensemble tree-based algorithms.

Conversely, KNN exhibited the largest normalized standard deviation (≈ 1.23) and was located farther from the reference observation, reflecting greater variability in the predicted values. Although AdaBoost produced a normalized Std. closer to unity (≈ 1.08), its larger centered RMSD indicates comparatively greater deviation from the experimental observations than the leading models. The increased RMSD observed for both KNN and AdaBoost is consistent with their broader prediction-error distributions presented in Fig. 4.

### Quantitative assessment of prediction models

**Fig. 7:**
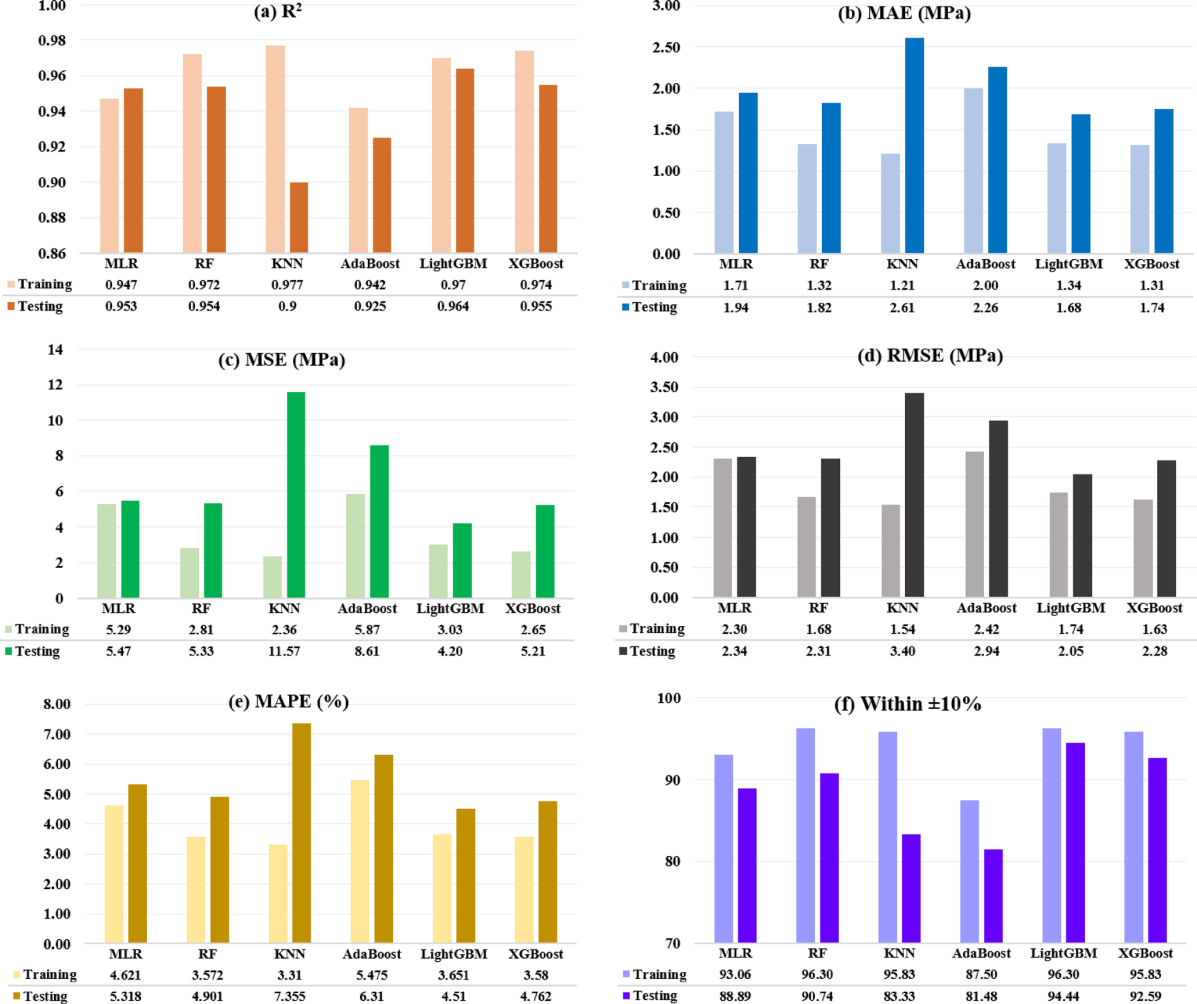
Bar charts of training and testing performance of each model.

A comprehensive ranking analysis was conducted to quantitatively evaluate and compare the predictive performance of the developed models. Figure 7 presents grouped bar charts illustrating the training and testing performance of each model based on six evaluation metrics: R², MAE, MSE, RMSE, MAPE, and the percentage of predictions within a ± 10% error margin. Subsequently, each model was ranked for every metric during both the training and testing phases, with ranks assigned from 1 (best performance) to 6 (worst performance), as summarized in Table 5. The overall ranking of each model was determined by summing its ranks across all evaluation metrics and both data subsets. Models with lower cumulative scores were considered the most reliable and accurate, reflecting superior predictive performance, whereas models with higher cumulative scores were regarded as less effective.

The ranking analysis for the training stage demonstrates that all models achieved high predictive performance, although clear differences were observed in their consistency across the six-evaluation metrics. KNN achieved the best overall performance with the lowest cumulative score **(8)**, ranking first in R², MAE, MSE, RMSE, and MAPE, while placing third for the percentage of predictions within the ± 10% error threshold. XGBoost ranked second with a cumulative score of **15**, consistently achieving second place in almost all performance indicators. RF followed closely with an overall score of **16**, exhibiting balanced performance across all metrics and obtaining the second-best result for the Within ± 10% criterion. LightGBM ranked fourth (score = **21**), despite sharing the highest percentage of predictions within the ± 10% error band (96.296%) with RF, reflecting its stable predictive performance during model training. The polynomial MLR model achieved an overall score of **31**, ranking fifth. Although it did not outperform the leading ensemble algorithms, it demonstrated competitive performance by consistently attaining fifth place across most evaluation metrics. In contrast, AdaBoost recorded the highest cumulative score (**35**), ranking sixth due to its comparatively larger prediction errors and lower overall consistency during the training phase.

The testing-stage ranking provides a more meaningful assessment of model generalization. LightGBM achieved the best overall performance with the lowest cumulative score (**6**), ranking first in R², MAE, MSE, RMSE, MAPE, and the percentage of predictions within the ± 10% error threshold (94.444%). XGBoost ranked second (score = **12**), maintaining second place across nearly all evaluation metrics, highlighting its excellent predictive stability on unseen data. Random Forest followed in third place with a cumulative score of **18**, consistently achieving third place in every evaluation criterion. Interestingly, the polynomial MLR model ranked fourth with an overall score of **24**, outperforming both AdaBoost and KNN during the testing phase. The model achieved competitive values across all performance metrics, indicating that the incorporation of polynomial feature expansion substantially enhanced its predictive capability and generalization performance. AdaBoost ranked fifth (score = **31**), while KNN obtained the highest cumulative score (35), ranking sixth due to its comparatively larger prediction errors and lower prediction consistency across the testing dataset.

Overall, the ranking analysis confirms the conclusions obtained from the previous statistical evaluations. While KNN demonstrated the strongest fitting performance during model training, LightGBM exhibited the most robust generalization capability and achieved the best overall performance on the independent testing dataset. XGBoost and Random Forest consistently maintained high rankings across both stages, whereas the polynomial MLR model provided competitive predictive performance despite its simpler mathematical formulation, reinforcing its suitability as a reliable baseline for comparison with advanced machine learning algorithms.

**Table:** Rank analysis for the implemented ML models.

Stage	Model	*R*²	MAE (MPa)	MSE (MPa)	RMSE (MPa)	MAPE (%)	Within ± 10%	Score	Overall rank
Training	MLR	(5)	(6)	(5)	(5)	(5)	(5)	31	5th
	RF	(3)	(3)	(3)	(3)	(2)	(2)	16	3rd
	**KNN** ^*^	(1)	(1)	(1)	(1)	(1)	(3)	8	**1st**
	AdaBoost	(6)	(5)	(6)	(6)	(6)	(6)	35	6th
	LightGBM	(4)	(4)	(4)	(4)	(4)	(1)	21	4th
	XGBoost	(2)	(2)	(2)	(2)	(3)	(4)	15	2nd
Testing	MLR	(4)	(4)	(4)	(4)	(4)	(4)	24	4th
	RF	(3)	(3)	(3)	(3)	(3)	(3)	18	3rd
	KNN	(6)	(6)	(6)	(6)	(6)	(5)	35	6th
	AdaBoost	(5)	(5)	(5)	(5)	(5)	(6)	31	5th
	**LightGBM** ^*^	(1)	(1)	(1)	(1)	(1)	(1)	6	**1st**
	XGBoost	(2)	(2)	(2)	(2)	(2)	(2)	12	2nd

* Note: The best-performing model in each stage is shown in bold.

### Interpretation of models

#### Features importance via SHAP

**Fig. 8:**
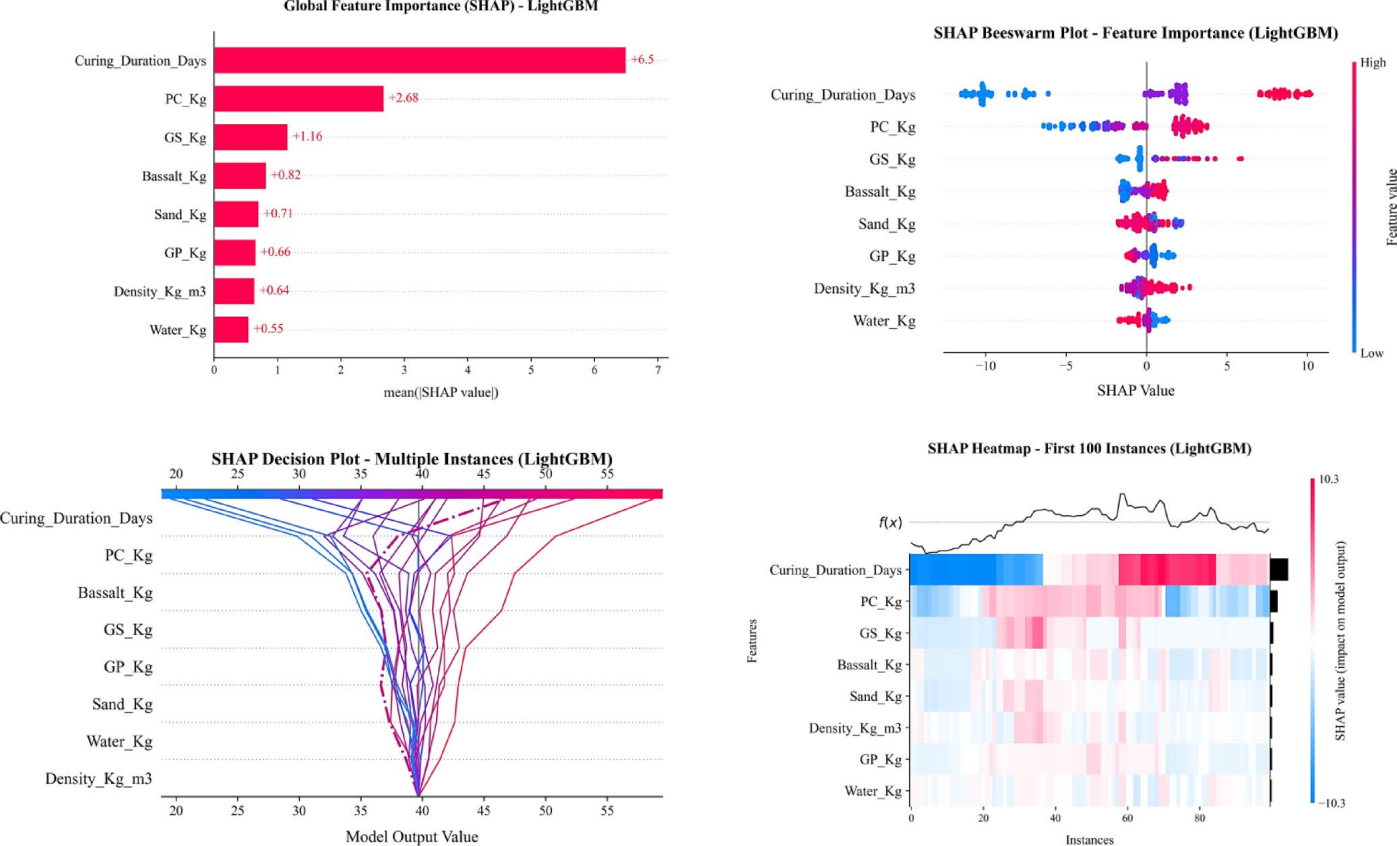
SHAP plots for best-performing model (LightGBM).

The primary objective of the sensitivity analysis is to quantitatively evaluate the relative influence of input parameters on the predicted compressive strength (CS) and to enhance the interpretability of the developed machine learning model. Based on the superior performance of the LightGBM model in the testing phase, it was selected for SHAP (Shapley Additive Explanations) analysis. SHAP provides both global and local interpretability by quantifying the contribution of each input variable to the model output. The results of this analysis are illustrated in Fig. 8 through multiple complementary visualizations.

The global SHAP feature importance plot clearly identifies the dominant role of input variables in influencing CS predictions. Among all features, curing duration exhibits the highest importance, with a mean SHAP value exceeding + 6.5, indicating its overwhelming contribution to strength development. This is followed by cement content (PC) with a mean contribution of approximately + 2.68, confirming its fundamental role in strength formation. Secondary influential parameters include glass sand (GS) ( ~ + 1.16), basalt ( ~ + 0.82), and sand ( ~ + 0.71), suggesting moderate contributions to the model output. In contrast, glass powder (GP), density, and water content show relatively lower importance values (ranging between + 0.55 and + 0.66), indicating a weaker but still notable influence on CS prediction. This ranking is consistent with established concrete behavior, where curing time and cementitious content govern strength development. The SHAP beeswarm plot provides deeper insight into both the magnitude and direction of feature effects. It reveals that higher curing durations (red points) consistently produce large positive SHAP values, significantly increasing predicted strength, while lower curing durations (blue points) result in negative contributions. A similar trend is observed for cement content, where higher PC values strongly enhance CS. Conversely, water content exhibits predominantly negative SHAP values, indicating that increased water reduces compressive strength due to higher porosity and weaker matrix structure. The distribution for GS shows both positive and negative contributions, reflecting its complex role depending on replacement level. Notably, GP tends to have slightly negative or marginal contributions, suggesting that excessive GP content may reduce strength beyond optimal levels. The relatively narrow spread of density and sand indicates limited variability in their influence.

The SHAP decision plot further illustrates how individual features cumulatively influence the model output across multiple instances. Starting from a base value of approximately 40 MPa, the contribution of each feature incrementally shifts the prediction. It is evident that curing duration consistently produces the largest upward shift, followed by cement content. In several instances, predictions increase from around 30–35 MPa to over 50–55 MPa, primarily driven by high curing duration and cement content. On the other hand, water and GP occasionally reduce the prediction, acting as limiting factors. The convergence of lines toward the final prediction values highlights the combined nonlinear interactions among variables captured by the LightGBM model. Finally, the SHAP heatmap provides a comprehensive visualization of feature contributions across multiple instances. The color gradient clearly demonstrates that curing duration dominates the prediction landscape, with strong red (positive) contributions in high-strength regions and blue (negative) contributions in low-strength cases. Cement content also shows consistent positive influence, while other variables exhibit more scattered and less intense contributions. The heatmap further confirms that most variability in predictions is driven by curing duration and cement, whereas other parameters contribute to fine-tuning the output. Overall, the SHAP analysis confirms that the LightGBM model not only achieves high predictive accuracy but also aligns with physical principles of concrete behavior. The dominance of curing duration and cement content, along with the negative influence of water, reflects well-established engineering knowledge, thereby enhancing the credibility, transparency, and practical applicability of the developed model.

#### PDP examination

Partial Dependence Plots (PDPs) were employed to further interpret the behavior of the developed LightGBM model by illustrating the relationship between individual input features and the predicted CS. PDPs provide a global interpretation by isolating the effect of a single variable while averaging out the influence of other features, thereby enabling a clear understanding of feature sensitivity^69^. In this study, one-dimensional PDPs (PDP-1D) were used to examine how variations in each input parameter influence the predicted output. The dashed line represents the average trend across the dataset, while the multiple light curves indicate variations across individual instances. This approach provides valuable insight into nonlinear relationships and ensures that the model behavior aligns with established engineering principles.

**Fig. 9:**
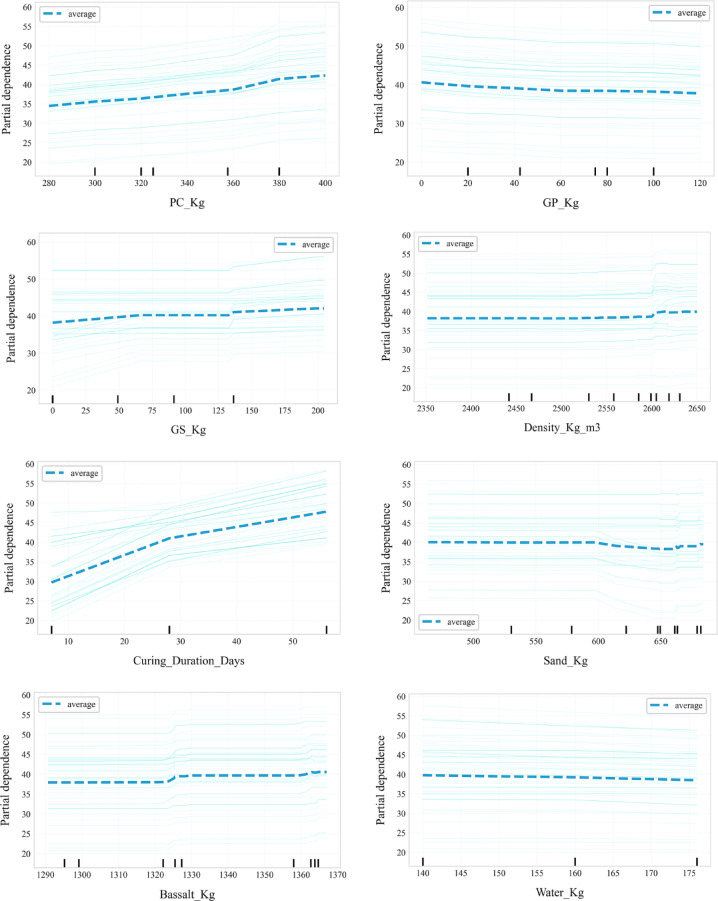
Partial dependence plots for studied features.

The PDP results in Fig. 9 reveal that PC exhibits a strong and nearly linear positive relationship with compressive strength. As PC increases from approximately 280 to 400 kg, the predicted CS rises steadily from around 35 MPa to over 42 MPa, reflecting the fundamental role of cement in hydration and strength development. Similarly, curing duration demonstrates the most pronounced influence among all variables, with predicted CS increasing significantly from approximately 30 MPa at early curing stages to nearly 48 MPa at extended curing periods. This steep upward trend highlights the critical role of continued hydration and microstructural densification in enhancing concrete strength.

In contrast, GP and water content exhibit negative relationships with compressive strength. Increasing GP content from 0 to 120 kg results in a slight decrease in predicted CS, suggesting that excessive replacement of cement with glass powder may reduce the availability of reactive cementitious material beyond optimal levels. Likewise, increasing water content from approximately 140 to 175 kg leads to a gradual reduction in strength, consistent with the well-established inverse relationship between water-to-cement ratio and compressive strength due to increased porosity and weaker matrix formation. The influence of GS, density, sand, and basalt is comparatively moderate. Glass sand shows a slight positive contribution, with predicted CS increasing modestly as GS content increases, although the step-like behavior of the curve suggests nonlinear interactions and possible threshold effects. Density exhibits a minor positive trend, indicating that higher density mixtures tend to produce slightly higher strength due to reduced voids. The PDP for basalt (coarse aggregate) also reflects a mild positive influence, likely associated with improved load transfer and aggregate interlock. Conversely, sand content demonstrates a slightly negative trend at higher values, suggesting that excessive fine aggregate may adversely affect the internal structure and reduce strength.

It should be noted that several input variables in the present dataset exhibit strong correlations due to the experimental mixture design. In particular, the (PC) and (GP) contents, as well as the sand and glass sand contents, are inherently linked through material replacement, resulting in high multicollinearity. Consequently, the SHAP and PDP analyses should be interpreted as model-based associations that describe each variable’s contribution to the model predictions rather than independent causal effects. The apparent influence of GP or GS may partially reflect the simultaneous reduction in cement or natural sand, respectively. Therefore, the feature importance and response trends identified in this study should be considered within the context of the adopted mixture design.

### Interactive GUI for prediction

**Fig. 10:**
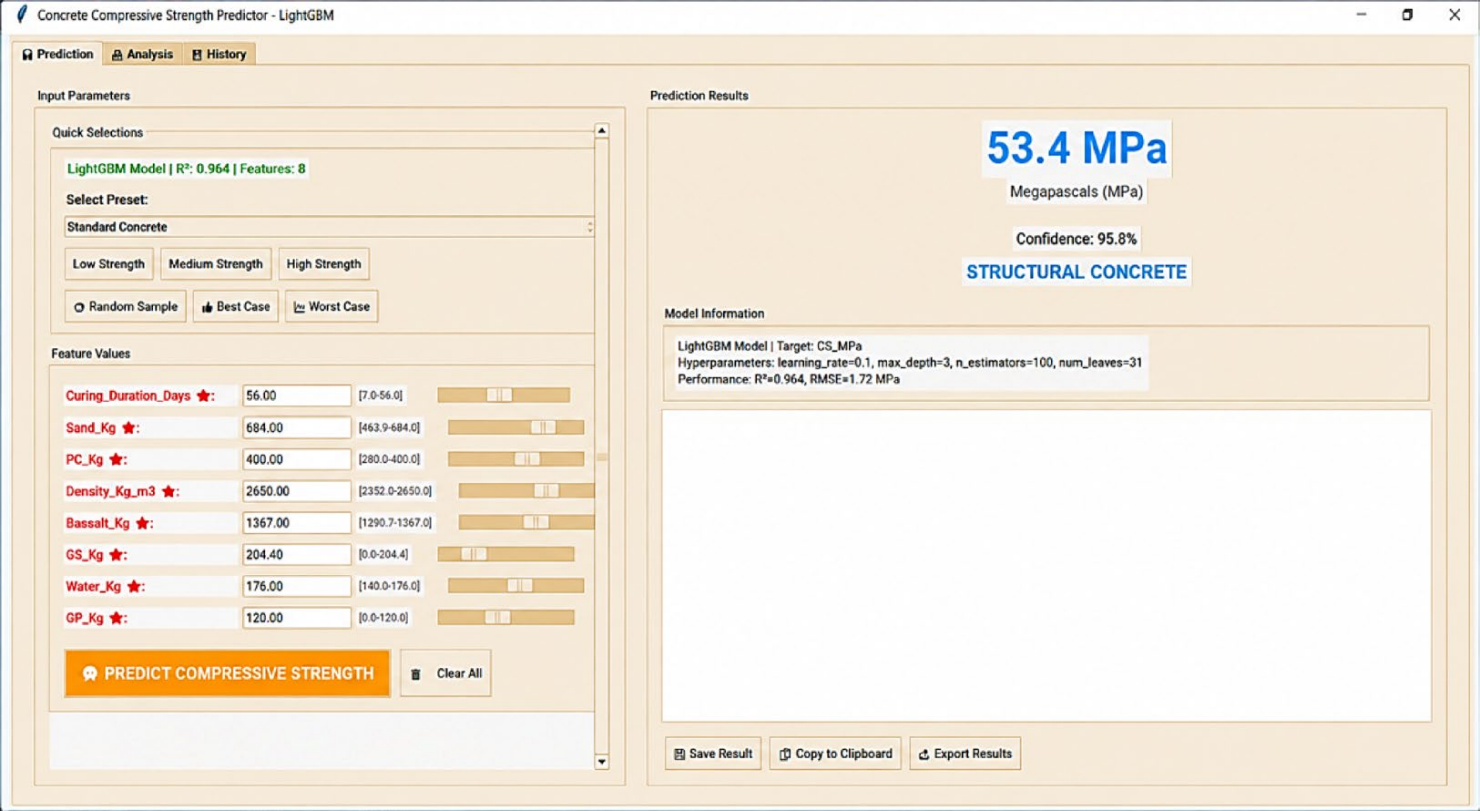
Predictive GUI designed for predicting the CS of sustainable concrete with waste glass materials.

This section presents a practical implementation of the developed machine learning model through a user-oriented application designed to facilitate its adoption in real-world engineering workflows. Despite the proven accuracy of ML models, their integration into routine design practice is often constrained by technical complexities related to data processing, model execution, and programming requirements^70^. To address these challenges, a Python-based Graphical User Interface (GUI) has been developed, incorporating the optimized LightGBM model for predicting the compressive strength (CS) of sustainable concrete incorporating glass powder (GP) and glass sand (GS). As illustrated in Fig. 10, the GUI provides a structured and intuitive environment that enables users to interact directly with the predictive model without requiring advanced programming knowledge.

The interface is organized into distinct sections, including input parameter controls and prediction output panels. Users can input key variables through editable fields. Upon execution, the system instantly provides the predicted compressive strength value (e.g., 53.4 MPa) along with a confidence level (e.g., 95.8%). The interface also displays key model information, including performance metrics (R² ≈ 0.964, RMSE ≈ 1.72 MPa) and optimized hyperparameters, ensuring transparency and reliability of predictions.

To promote accessibility and reproducibility, the developed GUI has been made available as an open-source tool via GitHub (https://github.com/SuhaibRasoolWani/CS-MPa-Eman). This allows researchers and practitioners to utilize, modify, and extend the application according to their specific needs. Overall, the proposed GUI represents a significant step toward bridging the gap between advanced ML models and practical engineering applications, enabling fast, reliable, and user-friendly prediction of concrete compressive strength in sustainable construction contexts.

### Comparison to established findings

To further assess the effectiveness of the proposed framework, the predictive performance of the developed LightGBM model was compared with representative ML studies reported in the literature (Table 6). These studies investigated CS prediction for various sustainable concrete mixtures incorporating different recycled or waste materials using a range of advanced ML techniques.

Ben Seghier et al.^46^ developed hybrid ML models combined with a marine predators algorithm (MPA) to predict the CS of waste-glass concrete. Among the investigated models, the LSSVR–MPA model achieved the highest accuracy (R² = 0.983 and RMSE = 2.447 MPa) using a dataset of 830 samples. Yehia et al.^71^ employed tree-based ensemble algorithms to predict the CS of concrete containing either waste glass (241 samples) or recycled concrete aggregate (319 samples), where XGBoost achieved the best performance with R² values of 0.920 and 0.876, respectively. Similarly, Sinkhonde et al.^72^ evaluated four ML algorithms, namely artificial neural network (ANN), RF, Decision tree (DT), and support vector machine (SVM) for concrete incorporating waste tyre rubber (WTR) and clay brick powder (CBP). They reported the RF model as the optimal model with R² = 0.889 and RMSE = 2.493 MPa. Bypour et al.^73^ investigated fly ash-based geopolymer concrete using six ML algorithms, where AdaBoost produced the best prediction accuracy (R² = 0.860, RMSE = 5.56 MPa, and MAE = 4.17 MPa).

In the present study, the optimized LightGBM model achieved R² = 0.964, RMSE = 2.05 MPa, and MAE = 1.68 MPa using a dataset of 270 observations. Although the hybrid LSSVR–MPA model reported by Ben Seghier et al.^46^ achieved a slightly higher coefficient of determination, the proposed LightGBM model produced a lower RMSE, indicating superior prediction accuracy in terms of absolute error. Overall, the proposed LightGBM model demonstrates competitive predictive capability compared with established models in the literature, confirming its suitability for accurate compressive strength prediction of sustainable concrete incorporating waste glass materials.

**Table 6:** Comparative analysis with previously developed models.

Refs.	Materials	Best model	Sample	*R*^2^	RMSE (MPa)	MAE (MPa)
^46^	Waste Glass (WG)	LSSVR-MPA	830	0.983	2.447	-
^71^	Waste Glass (WG)	XGBoost	241	0.920	2.67	1.277
^71^	Recycled concrete aggregate (RCA)	XGBoost	319	0.876	2.12	0.953
^72^	Waste Tyre rubber (WTR) and Clay brick powder (CBP)	RF	86	0.889	2.493	1.886
^73^	Fly ash based-Geopolymer	AdaBoost	161	0.86	5.56	4.17
**Current study**	Glass powder (GP) and glass sand (GS).	LightGBM	270	0.964	2.05	1.68

## Conclusions

This study presents a comprehensive Explainable Machine Learning (XML) framework for predicting the compressive strength (CS) of sustainable concrete incorporating waste glass materials. A total of 270 experimental observations were used to develop and validate six predictive models, including Random Forest (RF), K-Nearest Neighbors (KNN), AdaBoost, LightGBM, XGBoost, and Multiple Linear Regression (MLR) as a baseline statistical model. The models were developed using eight input parameters, namely Portland cement (PC), glass powder (GP), glass sand (GS), sand, basalt, water content, density, and curing duration. A 70/30 mixture-level data splitting strategy was adopted to eliminate potential data leakage and provide a more rigorous assessment of model generalization. The main findings are summarized as follows:

1. All developed ML models demonstrated satisfactory predictive capability, with ensemble learning algorithms outperforming the remaining approaches. Among them, LightGBM achieved the best overall performance on the testing dataset (R² = 0.964, RMSE = 2.05 MPa, and MAE = 1.68 MPa), with approximately 94.44% of the predictions falling within ± 10% of the measured compressive strength. XGBoost ranked second, demonstrating comparable predictive accuracy.
2. RF ranked third, achieving R² = 0.954, RMSE = 2.31 MPa, MAE = 1.82 MPa, and 90.74% of predictions within the ± 10% error range, confirming its strong predictive capability and robustness.
3. MLR provided a competitive baseline despite its simpler linear formulation, achieving a testing R² = 0.953 and MAE = 1.94 MPa, with 88.89% of predictions falling within the ± 10% error range. In contrast, KNN and AdaBoost exhibited higher prediction errors (i.e., RMSE and MAE up to 3.40 MPA and 2.60 MPa, respectively), particularly when evaluated on previously unseen mixture designs.
4. SHAP and PDP analyses enhanced the interpretability of the developed model by identifying curing duration and cement content as the most influential parameters governing compressive strength. Water content and glass powder exhibited negative relationships with CS, whereas GS, density, and aggregate contents (Basalt and Sand) showed moderate positive contributions. These explainability results should be interpreted as model-based associations within the investigated experimental domain.
5. To improve the practical applicability of the developed framework, the best-performing LightGBM model was successfully deployed into a user-friendly Python-based graphical user interface (GUI), enabling rapid and efficient prediction of compressive strength for sustainable concrete mixtures.
6. A comparison with previously published ML studies on sustainable concrete containing recycled and waste materials demonstrated that the proposed LightGBM model provides competitive predictive performance, achieving one of the lowest reported RMSE and MAE values.

## Limitations and future work

Despite the promising predictive performance and interpretability of the proposed framework, several limitations should be acknowledged. First, the developed models were trained using a relatively moderate dataset comprising 270 compressive strength measurements, which may limit their ability to capture the full variability associated with different raw material sources, mixture proportions, curing conditions, and environmental exposures. Expanding the database with more diverse experimental data would further improve the robustness and generalization capability of the developed models.

Second, the scope of this study is limited to the prediction of compressive strength, which, although one of the most important engineering properties, is insufficient to comprehensively characterize the performance of sustainable concrete. Other mechanical properties, including tensile and flexural strength, elastic modulus to support a more comprehensive material design framework.

Finally, the explainable AI analyses should be interpreted with caution because several input variables exhibit strong multicollinearity resulting from the experimental replacement strategy. In particular, the contents of Portland cement (PC) and glass powder (GP), as well as natural sand and glass sand (GS), are inherently linked through material substitution. Consequently, the SHAP and PDP results represent model-based associations rather than independent causal relationships. Future studies may improve the interpretability of explainable ML models by employing replacement ratios (e.g., GP replacement ratio and GS replacement ratio), water-to-binder ratio, or other independent mixture descriptors.

## Data Availability

The datasets used and analyzed during the current research are available from the corresponding author upon reasonable request.

## Appendix

See Table 7.

**Table 7:** Experimental mixtures and compressive strength dataset.

Mix ID	PC (Kg/m^3^)	GP (Kg/m^3^)	GS (Kg/m^3^)	Density (Kg/m^3^)	Sand (Kg/m^3^)	Basalt (Kg/m^3^)	Water (Kg/m^3^)	Curing Duration (Days)	CS (MPa)
**1**	400	0	0	2604	684	1367	140	7	33.570
	400	0	0	2604	684	1367	140	7	34.690
	400	0	0	2604	684	1367	140	7	33.730
	400	0	0	2604	684	1367	140	28	49.560
	400	0	0	2604	684	1367	140	28	45.900
	400	0	0	2604	684	1367	140	28	51.540
	400	0	0	2604	684	1367	140	56	59.700
	400	0	0	2604	684	1367	140	56	57.800
	400	0	0	2604	684	1367	140	56	58.890
**2**	380	20	0	2580	682.8	1365.7	140	7	32.25
	380	20	0	2580	682.8	1365.7	140	7	26.9
	380	20	0	2580	682.8	1365.7	140	7	31
	380	20	0	2580	682.8	1365.7	140	28	43.4
	380	20	0	2580	682.8	1365.7	140	28	42
	380	20	0	2580	682.8	1365.7	140	28	46.7
	380	20	0	2580	682.8	1365.7	140	56	54.4
	380	20	0	2580	682.8	1365.7	140	56	54.25
	380	20	0	2580	682.8	1365.7	140	56	53.3
**3**	360	40	0	2467	682	1364.2	140	7	27.96
	360	40	0	2467	682	1364.2	140	7	29.25
	360	40	0	2467	682	1364.2	140	7	28.29
	360	40	0	2467	682	1364.2	140	28	42.94
	360	40	0	2467	682	1364.2	140	28	41.71
	360	40	0	2467	682	1364.2	140	28	41.35
	360	40	0	2467	682	1364.2	140	56	53.38
	360	40	0	2467	682	1364.2	140	56	52.51
	360	40	0	2467	682	1364.2	140	56	53.11
**4**	340	60	0	2558	681.3	1362.7	140	7	26.63
	340	60	0	2558	681.3	1362.7	140	7	29.16
	340	60	0	2558	681.3	1362.7	140	7	26.1
	340	60	0	2558	681.3	1362.7	140	28	43.28
	340	60	0	2558	681.3	1362.7	140	28	40.39
	340	60	0	2558	681.3	1362.7	140	28	39.94
	340	60	0	2558	681.3	1362.7	140	56	52.68
	340	60	0	2558	681.3	1362.7	140	56	49.15
	340	60	0	2558	681.3	1362.7	140	56	50.57
**5**	320	80	0	2650	680.6	1361.2	140	7	24.24
	320	80	0	2650	680.6	1361.2	140	7	24.5
	320	80	0	2650	680.6	1361.2	140	7	26.27
	320	80	0	2650	680.6	1361.2	140	28	40.32
	320	80	0	2650	680.6	1361.2	140	28	40.28
	320	80	0	2650	680.6	1361.2	140	28	37.9
	320	80	0	2650	680.6	1361.2	140	56	48.64
	320	80	0	2650	680.6	1361.2	140	56	46.34
	320	80	0	2650	680.6	1361.2	140	56	47.51
**6**	300	100	0	2586	680	1359.7	140	7	23.58
	300	100	0	2586	680	1359.7	140	7	23.94
	300	100	0	2586	680	1359.7	140	7	26.58
	300	100	0	2586	680	1359.7	140	28	38.83
	300	100	0	2586	680	1359.7	140	28	40.11
	300	100	0	2586	680	1359.7	140	28	35.07
	300	100	0	2586	680	1359.7	140	56	45.9
	300	100	0	2586	680	1359.7	140	56	42.78
	300	100	0	2586	680	1359.7	140	56	44.22
**7**	280	120	0	2636	679	1358	140	7	23.05
	280	120	0	2636	679	1358	140	7	23.67
	280	120	0	2636	679	1358	140	7	19.28
	280	120	0	2636	679	1358	140	28	37.62
	280	120	0	2636	679	1358	140	28	35.66
	280	120	0	2636	679	1358	140	28	34.73
	280	120	0	2636	679	1358	140	56	38.8
	280	120	0	2636	679	1358	140	56	42.12
	280	120	0	2636	679	1358	140	56	43.29
**8**	380	20	68.2	2631	614.1	1364.7	140	7	42.300
	380	20	68.2	2631	614.1	1364.7	140	7	37.300
	380	20	68.2	2631	614.1	1364.7	140	7	40.400
	380	20	68.2	2631	614.1	1364.7	140	28	45.260
	380	20	68.2	2631	614.1	1364.7	140	28	48.100
	380	20	68.2	2631	614.1	1364.7	140	28	44.630
	380	20	68.2	2631	614.1	1364.7	140	56	55.440
	380	20	68.2	2631	614.1	1364.7	140	56	57.170
	380	20	68.2	2631	614.1	1364.7	140	56	54.190
**9**	380	20	136.4	2617	545.5	1363.8	140	7	40.07
	380	20	136.4	2617	545.5	1363.8	140	7	43.65
	380	20	136.4	2617	545.5	1363.8	140	7	43.47
	380	20	136.4	2617	545.5	1363.8	140	28	49.5
	380	20	136.4	2617	545.5	1363.8	140	28	46.92
	380	20	136.4	2617	545.5	1363.8	140	28	47.58
	380	20	136.4	2617	545.5	1363.8	140	56	59.23
	380	20	136.4	2617	545.5	1363.8	140	56	57.09
	380	20	136.4	2617	545.5	1363.8	140	56	54.68
**10**	380	20	204.4	2619	477	1362.9	140	7	46.06
	380	20	204.4	2619	477	1362.9	140	7	42.72
	380	20	204.4	2619	477	1362.9	140	7	45.02
	380	20	204.4	2619	477	1362.9	140	28	51.27
	380	20	204.4	2619	477	1362.9	140	28	48.64
	380	20	204.4	2619	477	1362.9	140	28	48.59
	380	20	204.4	2619	477	1362.9	140	56	57.4
	380	20	204.4	2619	477	1362.9	140	56	60.76
	380	20	204.4	2619	477	1362.9	140	56	57.34
**11**	400	0	0	2544	665.8	1329.7	160	7	27.380
	400	0	0	2544	665.8	1329.7	160	7	34.100
	400	0	0	2544	665.8	1329.7	160	7	28.520
	400	0	0	2544	665.8	1329.7	160	28	45.120
	400	0	0	2544	665.8	1329.7	160	28	45.670
	400	0	0	2544	665.8	1329.7	160	28	47.210
	400	0	0	2544	665.8	1329.7	160	56	59.770
	400	0	0	2544	665.8	1329.7	160	56	55.460
	400	0	0	2544	665.8	1329.7	160	56	57.270
**12**	380	20	0	2516	664.1	1328.2	160	7	27.51
	380	20	0	2516	664.1	1328.2	160	7	27.49
	380	20	0	2516	664.1	1328.2	160	7	29
	380	20	0	2516	664.1	1328.2	160	28	42.88
	380	20	0	2516	664.1	1328.2	160	28	39.14
	380	20	0	2516	664.1	1328.2	160	28	42.48
	380	20	0	2516	664.1	1328.2	160	56	49.31
	380	20	0	2516	664.1	1328.2	160	56	50.07
	380	20	0	2516	664.1	1328.2	160	56	50.62
**13**	360	40	0	2478	663.3	1326.7	160	7	25.59
	360	40	0	2478	663.3	1326.7	160	7	26.03
	360	40	0	2478	663.3	1326.7	160	7	26.38
	360	40	0	2478	663.3	1326.7	160	28	38.51
	360	40	0	2478	663.3	1326.7	160	28	39.15
	360	40	0	2478	663.3	1326.7	160	28	39.34
	360	40	0	2478	663.3	1326.7	160	56	45.51
	360	40	0	2478	663.3	1326.7	160	56	45.43
	360	40	0	2478	663.3	1326.7	160	56	44.06
**14**	340	60	0	2453	662.6	1325.2	160	7	24.97
	340	60	0	2453	662.6	1325.2	160	7	26.07
	340	60	0	2453	662.6	1325.2	160	7	23.95
	340	60	0	2453	662.6	1325.2	160	28	36.43
	340	60	0	2453	662.6	1325.2	160	28	39.78
	340	60	0	2453	662.6	1325.2	160	28	39
	340	60	0	2453	662.6	1325.2	160	56	44.43
	340	60	0	2453	662.6	1325.2	160	56	45.26
	340	60	0	2453	662.6	1325.2	160	56	41.41
**15**	320	80	0	2442	661.9	1323.7	160	7	20.93
	320	80	0	2442	661.9	1323.7	160	7	23.25
	320	80	0	2442	661.9	1323.7	160	7	21.82
	320	80	0	2442	661.9	1323.7	160	28	38.46
	320	80	0	2442	661.9	1323.7	160	28	35.93
	320	80	0	2442	661.9	1323.7	160	28	37.21
	320	80	0	2442	661.9	1323.7	160	56	42.38
	320	80	0	2442	661.9	1323.7	160	56	38.82
	320	80	0	2442	661.9	1323.7	160	56	41.8
**16**	300	100	0	2368	661.1	1322.2	160	7	20.57
	300	100	0	2368	661.1	1322.2	160	7	21.89
	300	100	0	2368	661.1	1322.2	160	7	18.14
	300	100	0	2368	661.1	1322.2	160	28	36.36
	300	100	0	2368	661.1	1322.2	160	28	33.52
	300	100	0	2368	661.1	1322.2	160	28	35.12
	300	100	0	2368	661.1	1322.2	160	56	37.11
	300	100	0	2368	661.1	1322.2	160	56	39.04
	300	100	0	2368	661.1	1322.2	160	56	42.35
**17**	280	120	0	2352	660.4	1320.7	160	7	18.12
	280	120	0	2352	660.4	1320.7	160	7	20.73
	280	120	0	2352	660.4	1320.7	160	7	18.14
	280	120	0	2352	660.4	1320.7	160	28	32
	280	120	0	2352	660.4	1320.7	160	28	34.76
	280	120	0	2352	660.4	1320.7	160	28	35.25
	280	120	0	2352	660.4	1320.7	160	56	35.25
	280	120	0	2352	660.4	1320.7	160	56	37.03
	280	120	0	2352	660.4	1320.7	160	56	38.12
**18**	380	20	66.4	2585	597.3	1327.3	160	7	39.620
	380	20	66.4	2585	597.3	1327.3	160	7	36.190
	380	20	66.4	2585	597.3	1327.3	160	7	41.190
	380	20	66.4	2585	597.3	1327.3	160	28	42.170
	380	20	66.4	2585	597.3	1327.3	160	28	45.310
	380	20	66.4	2585	597.3	1327.3	160	28	44.820
	380	20	66.4	2585	597.3	1327.3	160	56	53.930
	380	20	66.4	2585	597.3	1327.3	160	56	49.290
	380	20	66.4	2585	597.3	1327.3	160	56	49.180
**19**	380	20	132.6	2619	530.5	1326.4	160	7	43
	380	20	132.6	2619	530.5	1326.4	160	7	39.69
	380	20	132.6	2619	530.5	1326.4	160	7	40.31
	380	20	132.6	2619	530.5	1326.4	160	28	47.9
	380	20	132.6	2619	530.5	1326.4	160	28	44.72
	380	20	132.6	2619	530.5	1326.4	160	28	43.88
	380	20	132.6	2619	530.5	1326.4	160	56	54.2
	380	20	132.6	2619	530.5	1326.4	160	56	51.83
	380	20	132.6	2619	530.5	1326.4	160	56	49.97
**20**	380	20	198.8	2635	463.9	1325.5	160	7	42.26
	380	20	198.8	2635	463.9	1325.5	160	7	45.05
	380	20	198.8	2635	463.9	1325.5	160	7	57.37
	380	20	198.8	2635	463.9	1325.5	160	28	44.6
	380	20	198.8	2635	463.9	1325.5	160	28	49.32
	380	20	198.8	2635	463.9	1325.5	160	28	45.44
	380	20	198.8	2635	463.9	1325.5	160	56	53.92
	380	20	198.8	2635	463.9	1325.5	160	56	54.01
	380	20	198.8	2635	463.9	1325.5	160	56	57.37
**21**	400	0	0	2629	649.8	1299.7	176	7	27.570
	400	0	0	2629	649.8	1299.7	176	7	28.820
	400	0	0	2629	649.8	1299.7	176	7	27.620
	400	0	0	2629	649.8	1299.7	176	28	42.190
	400	0	0	2629	649.8	1299.7	176	28	42.860
	400	0	0	2629	649.8	1299.7	176	28	42.450
	400	0	0	2629	649.8	1299.7	176	56	53.830
	400	0	0	2629	649.8	1299.7	176	56	51.980
	400	0	0	2629	649.8	1299.7	176	56	50.180
**22**	380	20	0	2450	649.1	1298.2	176	7	27.23
	380	20	0	2450	649.1	1298.2	176	7	26.78
	380	20	0	2450	649.1	1298.2	176	7	25.49
	380	20	0	2450	649.1	1298.2	176	28	40.53
	380	20	0	2450	649.1	1298.2	176	28	40.68
	380	20	0	2450	649.1	1298.2	176	28	41.79
	380	20	0	2450	649.1	1298.2	176	56	45.93
	380	20	0	2450	649.1	1298.2	176	56	45.32
	380	20	0	2450	649.1	1298.2	176	56	46.75
**23**	360	40	0	2599	648.3	1296.7	176	7	25.09
	360	40	0	2599	648.3	1296.7	176	7	25.94
	360	40	0	2599	648.3	1296.7	176	7	24.87
	360	40	0	2599	648.3	1296.7	176	28	36.69
	360	40	0	2599	648.3	1296.7	176	28	39.53
	360	40	0	2599	648.3	1296.7	176	28	37.78
	360	40	0	2599	648.3	1296.7	176	56	43.85
	360	40	0	2599	648.3	1296.7	176	56	44.28
	360	40	0	2599	648.3	1296.7	176	56	45.37
**24**	340	60	0	2553	647.6	1295.2	176	7	20.4
	340	60	0	2553	647.6	1295.2	176	7	21.87
	340	60	0	2553	647.6	1295.2	176	7	24.94
	340	60	0	2553	647.6	1295.2	176	28	38.38
	340	60	0	2553	647.6	1295.2	176	28	37.14
	340	60	0	2553	647.6	1295.2	176	28	35.49
	340	60	0	2553	647.6	1295.2	176	56	40.1
	340	60	0	2553	647.6	1295.2	176	56	43.48
	340	60	0	2553	647.6	1295.2	176	56	42.42
**25**	320	80	0	2600	646.9	1293.7	176	7	18.65
	320	80	0	2600	646.9	1293.7	176	7	22.82
	320	80	0	2600	646.9	1293.7	176	7	20.93
	320	80	0	2600	646.9	1293.7	176	28	36.88
	320	80	0	2600	646.9	1293.7	176	28	33.74
	320	80	0	2600	646.9	1293.7	176	28	37.39
	320	80	0	2600	646.9	1293.7	176	56	39.58
	320	80	0	2600	646.9	1293.7	176	56	38.84
	320	80	0	2600	646.9	1293.7	176	56	41.58
**26**	300	100	0	2605	646.1	1292.2	176	7	18.72
	300	100	0	2605	646.1	1292.2	176	7	16.97
	300	100	0	2605	646.1	1292.2	176	7	22.82
	300	100	0	2605	646.1	1292.2	176	28	34.28
	300	100	0	2605	646.1	1292.2	176	28	35.41
	300	100	0	2605	646.1	1292.2	176	28	30.81
	300	100	0	2605	646.1	1292.2	176	56	36.07
	300	100	0	2605	646.1	1292.2	176	56	39.46
	300	100	0	2605	646.1	1292.2	176	56	40.57
**27**	280	120	0	2618	645.4	1290.7	176	7	19.47
	280	120	0	2618	645.4	1290.7	176	7	19.54
	280	120	0	2618	645.4	1290.7	176	7	15.59
	280	120	0	2618	645.4	1290.7	176	28	34.72
	280	120	0	2618	645.4	1290.7	176	28	29.82
	280	120	0	2618	645.4	1290.7	176	28	31.47
	280	120	0	2618	645.4	1290.7	176	56	33.72
	280	120	0	2618	645.4	1290.7	176	56	34.8
	280	120	0	2618	645.4	1290.7	176	56	37.68
**28**	380	20	68.2	2540	614.1	1364.7	176	7	32.430
	380	20	68.2	2540	614.1	1364.7	176	7	33.730
	380	20	68.2	2540	614.1	1364.7	176	7	35.850
	380	20	68.2	2540	614.1	1364.7	176	28	41.470
	380	20	68.2	2540	614.1	1364.7	176	28	40.400
	380	20	68.2	2540	614.1	1364.7	176	28	45.030
	380	20	68.2	2540	614.1	1364.7	176	56	48.430
	380	20	68.2	2540	614.1	1364.7	176	56	47.060
	380	20	68.2	2540	614.1	1364.7	176	56	45.520
**29**	380	20	136.4	2594	545.5	1363.8	176	7	38.56
	380	20	136.4	2594	545.5	1363.8	176	7	37.1
	380	20	136.4	2594	545.5	1363.8	176	7	35.94
	380	20	136.4	2594	545.5	1363.8	176	28	43.8
	380	20	136.4	2594	545.5	1363.8	176	28	42.32
	380	20	136.4	2594	545.5	1363.8	176	28	46.19
	380	20	136.4	2594	545.5	1363.8	176	56	50
	380	20	136.4	2594	545.5	1363.8	176	56	49.02
	380	20	136.4	2594	545.5	1363.8	176	56	45.88
**30**	380	20	204.4	2527	477	1362.9	176	7	37.24
	380	20	204.4	2527	477	1362.9	176	7	41.02
	380	20	204.4	2527	477	1362.9	176	7	37.85
	380	20	204.4	2527	477	1362.9	176	28	45.32
	380	20	204.4	2527	477	1362.9	176	28	44.42
	380	20	204.4	2527	477	1362.9	176	28	47.96
	380	20	204.4	2527	477	1362.9	176	56	48.27
	380	20	204.4	2527	477	1362.9	176	56	52.39
	380	20	204.4	2527	477	1362.9	176	56	47.84
